# Ca^2+^-Dependent Effects of the Selenium-Sorafenib Nanocomplex on Glioblastoma Cells and Astrocytes of the Cerebral Cortex: Anticancer Agent and Cytoprotector

**DOI:** 10.3390/ijms24032411

**Published:** 2023-01-26

**Authors:** Elena G. Varlamova, Venera V. Khabatova, Sergey V. Gudkov, Egor A. Turovsky

**Affiliations:** 1 Institute of Cell Biophysics of the Russian Academy of Sciences, Federal Research Center “Pushchino Scientific Center for Biological Research of the Russian Academy of Sciences”, 142290 Pushchino, Russia; 2 Prokhorov General Physics Institute of the Russian Academy of Sciences, 38 Vavilove st., 119991 Moscow, Russia; 3 Institute of Biology and Biomedicine, Lobachevsky State University of Nizhny Novgorod, 23 Gagarin Ave., 603022 Nizhny Novgorod, Russia

**Keywords:** sorafenib, selenium nanoparticles, selenium–sorafenib nanocomplex, glioblastoma, astrocytes, apoptosis, ER stress, necrosis, calcium, protein kinases

## Abstract

Despite the fact that sorafenib is recommended for the treatment of oncological diseases of the liver, kidneys, and thyroid gland, and recently it has been used for combination therapy of brain cancer of various genesis, there are still significant problems for its widespread and effective use. Among these problems, the presence of the blood–brain barrier of the brain and the need to use high doses of sorafenib, the existence of mechanisms for the redistribution of sorafenib and its release in the brain tissue, as well as the high resistance of gliomas and glioblastomas to therapy should be considered the main ones. Therefore, there is a need to create new methods for delivering sorafenib to brain tumors, enhancing the therapeutic potential of sorafenib and reducing the cytotoxic effects of active compounds on the healthy environment of tumors, and ideally, increasing the survival of healthy cells during therapy. Using vitality tests, fluorescence microscopy, and molecular biology methods, we showed that the selenium-sorafenib (SeSo) nanocomplex, at relatively low concentrations, is able to bypass the mechanisms of glioblastoma cell chemoresistance and to induce apoptosis through Ca^2+^-dependent induction of endoplasmic reticulum stress, changes in the expression of selenoproteins and selenium-containing proteins, as well as key kinases-regulators of oncogenicity and cell death. Selenium nanoparticles (SeNPs) also have a high anticancer efficacy in glioblastomas, but are less selective, since SeSo in cortical astrocytes causes a more pronounced activation of the cytoprotective pathways.

## 1. Introduction

Glioblastomas are the most aggressive forms of brain cancer in adults and are difficult to treat, characterized with high invasiveness and resistance to known drugs. As a result, surgery followed by radiotherapy and chemotherapy results in 2 and 5-year patient survival rates of 27% and 5%, respectively [1,2,3]. The median survival in patients with anaplastic astrocytoma is about 30 months, while it is less than 15 months in the case of glioblastoma multiforme under standard care treatment. Gliomas are characterized with an aggressive proliferation and growth, as well as diffuse infiltration into the adjacent parenchyma of the brain. They are also highly resistant to apoptosis during chemotherapy [4,5]. These phenomena are associated with mutations or increased expression of pro-survival kinases—Ras-Raf-MEK-ERK in glioma cells [6].

Sorafenib is recommended by U.S. Food and Drug Administration as a first-line drug for the treatment of liver and kidney cancer. Sorafenib is a multikinase inhibitor [7] and acts on a cascade of key protein kinases: endothelial growth factor receptor (VEGFR) kinases, platelet-derived growth factor receptor (PDGFR) kinases, MAP kinase signaling (i.e., ERK, JNK, and p38), STAT3-associated IL-6, and NF-kB-linked COX-2 levels [8,9], but at the same time it contributes to the activation of fibrosarcoma (RAF) kinases [8]. Since sorafenib is an inhibitor of a wide range of kinases, there is more and more evidence of its effective action in brain tumors of various origins [10]. However, there are ambiguous data on the effects of sorafenib on cognitive functions depending on the state of the body, which should be taken into account. Conflicting effects of sorafenib on cognitive/synaptic function have been reported. In APPswe mice (a mouse model of AD), sorafenib treatment modulates the neuroinflammatory responses to restore working memory [11]. On the contrary, negative effects of sorafenib on cognitive function via disruption of metabonomic pathways have been observed in cancer patients [12,13]. In addition, sorafenib has a whole range of objective disadvantages—low bioavailability due to poor solubility and high metabolism in tissues, the need for high doses for cancer therapy and, as a result, the appearance of numerous side effects on healthy organs and tissues. Among the side effects of therapeutic doses of sorafenib, the appearance of skin diseases, diarrhea, hypertension, fatigue, hand-foot syndrome, bleeding, arterial thromboembolic events, etc., should be mentioned [14]. In the treatment of brain cancer, the problem of using sorafenib is exacerbated due to the presence of the blood–brain barrier, which requires the use of even higher concentrations of this anticancer agent, compared with the treatment of cancer of other organs.

Selenium nanoparticles (SeNPs) have shown themselves to be a very effective anticancer agent, dose-dependently inducing apoptosis through Ca^2+^-dependent and Ca^2+^-independent mechanisms [15,16,17]. At the same time, the pleiotropic effect of nanoselenium is well known, which consists in the induction of cell death of cancer cells, on the one hand, and the strengthening of the protective mechanisms of neurons and astrocytes of the brain, on the other hand [18,19,20]. Since nanoselenium has the lowest cytotoxicity, compared to nanoparticles of a different nature [21], constructs where SeNPs act as a carrier of active agents for the treatment of a wide range of diseases begin to appear much more often [22,23,24,25]. Another uniqueness of SeNPs is their high ability to penetrate the blood–brain barrier and reach brain cells [26,27], while sorafenib penetrates the blood–brain barrier even at high doses [28], but in the case of glioblastomas, sorafenib efflux occurs. It has been shown that ATP-binding cassette transporters P-glycoprotein and breast cancer resistance protein (BCRP) together restrict the brain distribution of sorafenib with BCRP playing a dominant role in the efflux of sorafenib at the blood–brain barrier [29]. Therefore, it is of interest to create nanocomplexes for the efficient delivery of sorafenib to brain tumors. Of the known sorafenib nanotransporters, lipid nanocapsules (LNC-SFN) have shown a high efficiency. Daily intraperitoneal injections of sorafenib (100 mg/kg) into mice with glioblastoma resulted in the inhibition of tumor-cell proliferation and lower levels of angiogenesis. However, a single injection of sorafenib encapsulated in lipid nanocapsules (LNC-SFN) at a concentration of 3.5 μg/mouse leads to more pronounced anticancer effects [30]. There is also evidence of enhancement of the effect of sorafenib by adding another active substance when the anti-glioma potential of sorafenib was enhanced by quercetin [31]. We have developed and tested on human hepatocellular carcinoma cells the selenium-sorafenib nanocomplex (SeSo)—the selenium nanoparticles 100 nm in size, on which sorafenib is adsorbed. It has been shown that SeSo has a more than 20% effective anticancer activity, compared to “naked” SeNPs and many times exceeds the effectiveness of sorafenib used at similar and higher concentrations [32]. In this regard, we assume that the doping of SeNPs with sorafenib will lead to the activation of apoptosis in glioblastomas and gliomas, due to the action of sorafenib-enhanced nanoselenium, but at the same time, the mechanisms of suppression of neuroinflammation in healthy brain tissue, due to sorafenib will be activated. In this case, the effect of high doses of sorafenib will be maximally reduced if it was used in its pure form, and not in the form of a nanocomplex.

## 2. Results

### 2.1. Effect of Sorafenib, Nanoselenium, and Selenium-Sorafenib Nanocomplex on the Ca^2+^ Signaling System of A-172 Glioblastoma Cells and Astrocytes of the Cerebral Cortex

**Figure 1:**
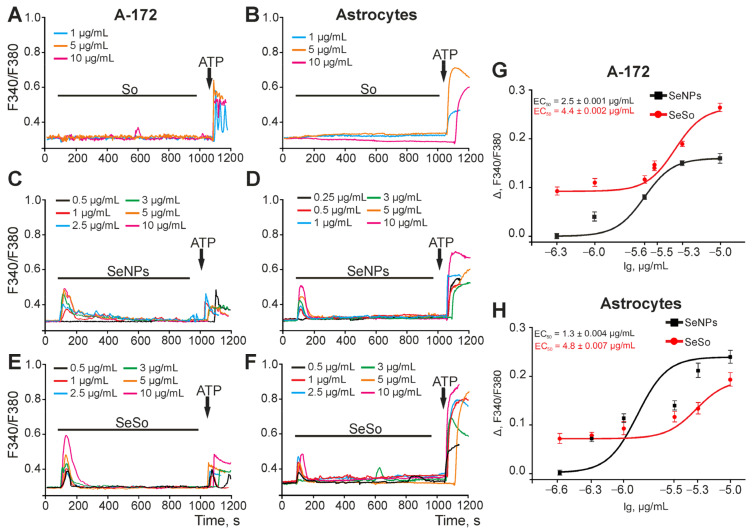
Activation of the Ca^2+^-responses of A-172 cells (**A**,**C**,**E**) and cortical astrocytes (**B**,**D**,**F**) by different concentrations of So, SeNPs, and SeSo. (**A**,**C**,**E**)—Application of various concentrations of So (**A**), SeNPs (**C**), and SeSo (**E**) induces the generation of Ca^2+^-responses in the A-172 cells. (**B**,**D**,**F**)—Application of various concentrations of So (**B**), SeNPs (**D**), and SeSo (**F**) induces the generation of Ca^2+^-responses in the cortical astrocytes. Ca^2+^-responses averaged over several tens of cells in one experiment are presented. (**G**,**H**)—Dependence of the amplitude of Ca^2+^ responses of A-172 cells (**G**) and astrocytes (**H**) on the growth concentration of SeNPs (black squares) and SeSo (red circles) and its approximation by a sigmoid function.

To record the [Ca^2+^]_i_ dynamics, the cells were loaded with a calcium-sensitive Fura-2 probe and, using fluorescence microscopy, the change in [Ca^2+^]_i_ was recorded upon application of sorafenib (So), naked selenium nanoparticles (SeNPs), and the selenium-sorafenib nanocomplex (SeSo) in A-172 glioblastoma cells or astrocytes of the cerebral cortex. Application of sorafenib at concentrations of 1, 3, and 10 μg/mL to A-172 cells (Figure 1A) or astrocytes (Figure 1B) did not cause the generation of Ca^2+^-responses. At the same time, the calcium signaling system of the cells was absolutely functional, and the addition of 10 μM ATP led to an increase in [Ca^2+^]_i_ through the activation of purinergic receptors and the mobilization of Ca^2+^ ions from the ER. Selenium nanoparticles (SeNPs) in glioblastoma cells dose-dependently induced an increase in [Ca^2+^]_i_ (Figure 1C) with EC50 = 2.5 ± 0.001 μg/mL (Figure 1G) and in astrocytes of the cerebral cortex (Figure 1D) with EC50 = 1.3 ± 0.004 μg /mL (Figure 1H). Ca^2+^-responses upon application of SeNPs in both cell types were transient and the level of [Ca^2+^]_i_ increases returned to the baseline level (before application of SeNPs), but on average, their amplitude was higher in astrocytes of the cerebral cortex (Figure 1D). Application of the selenium-sorafenib nanocomplex (SeSo) also led to the generation of Ca^2+^ responses by A-172 glioblastoma cells (Figure 1E) and cortical astrocytes (Figure 1F), the Ca^2+^-responses were transient with the return of [Ca^2+^]_i_ to the baseline level. At the same time, the EC50 values for A-172 cells and astrocytes of the cerebral cortex were almost similar and amounted to 4.4 ± 0.002 μg/mL (Figure 1G) and 4.8 ± 0.007 μg/mL (Figure 1H), respectively. However, if we compare the effect of “naked” SeNPs and SeSo on A-172 glioblastoma cells, it can be seen that the sigmoid curve approximating the amplitudes of Ca^2+^-responses to the addition of various concentrations of SeSo lies higher than the curve approximating the Ca^2+^ responses to SeNPs (Figure 1G), which indicates a higher sensitivity of the Ca^2+^ signaling system of A-172 cells to the SeSo nanocomplex. At the same time, astrocytes of the cerebral cortex also began to respond with Ca^2+^ responses to the addition of even low concentrations of SeSo (Figure 1H), compared to SeNPs. However, in the range of high concentrations (5–10 μg/mL), “naked” SeNPs caused greater amplitude of Ca^2+^-responses, compared with SeSo. In general, for astrocytes, the sigmoid curve approximating the amplitudes of Ca^2+^-responses on SeSo is located below the curve for “naked” SeNPs (Figure 1H).

Thus, sorafenib at the studied concentrations did not cause rapid reactions in the form of an increase in [Ca^2+^]_i_ in A-172 glioblastoma cells and astrocytes of the cerebral cortex, which may indicate the absence of a fast signaling component of sorafenib associated with intracellular calcium signaling. At the same time, the selenium-sorafenib nanocomplex was more pronounced and selectively activated the Ca^2+^ signaling system of A-172 glioblastoma cells, compared to “naked” SeNPs, causing higher amplitude Ca^2+^ responses. Comparing glioblastoma cells with cortical astrocytes, astrocytes are more sensitive to the application of SeNPs, having a lower EC50, compared to glioblastoma cells. However, in terms of sensitivity and EC50 value for the selenium-sorafenib nanocomplex, astrocytes and glioblastoma cells practically did not differ. «Naked»SeNPs and the SeSo nanocomplex inhibited the proliferation of A-172 cells in a dose-dependent manner without any significant difference between them.

### 2.2. Effect of Sorafenib, Nanoselenium, and Selenium-Sorafenib Nanocomplex on the Induction of Cell Death

**Figure 2:**
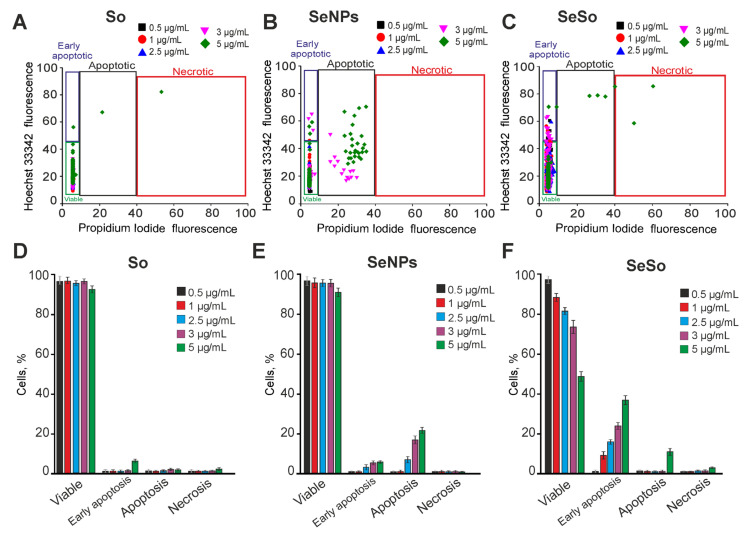
Effect of 24-h incubation of A-172 cells with various concentrations of sorafenib (So), selenium nanoparticles (SeNPs), and selenium-sorafenib nanocomplex (SeSo). (**A**–**C**)–Cytograms demonstrating the viability of A-172 cells after 24-h pre-incubation with different concentrations of So (**A**), SeNPs (**B**), and SeSo (**C**). X-axis: the intensity of PI fluorescence; Y-axis: the intensity of Hoechst 33342 fluorescence. Cells were stained with the probes after 24-h incubation with different concentrations of compounds. (**D**–**F**)—The comparison of the effect of different concentrations of So, SeNPs, and SeSo on A-172 cell survival after 24-h pre-incubation. Panels (**D**–**F**) is a calculation (technical replicate) of the data (mean ± SE) presented in panels (**A**–**C**), performed in four repetitions, *n* = 4. Cell images are shown in Supplementary Figures S1–S3.

Pre-incubation of A-172 human glioblastoma cells for 24 h with sorafenib at concentrations of 0.5–5 μg/mL does not cause apoptosis and necrotic death, only a concentration of 5 μg/mL induces the death of single cells (Figure 2A,D; Supplementary Figure S1). Previously, we showed that selenium nanoparticles (SeNPs) had an anticancer effect in various human tumor cell lines [16]. The addition of SeNPs to A-172 cells at concentrations of 0.5–2.5 μg/mL after 24 h did not cause a significant increase in apoptotic cells, but an increase of SeNPs concentration to 3–5 μg/mL led to an increase of cell percentage at the predominantly late stages of apoptosis (in 12–23% of cells) (Figure 2B,E; Supplementary Figure S2). Selenium nanoparticles doped with sorafenib (SeSo) at concentrations of 1–5 μg/mL already after 24 h dose-dependently caused the induction of early stages of apoptosis in 12–38% of A-172 cells, without massive activation of late stages of apoptosis or necrosis (Figure 2C,F; Supplementary Figure S3). Following exposure to 5 μg/mL SeSo, only single cells were registered at the late stages of apoptosis and with necrosis (Figure 2C,F; Supplementary Figure S3).

**Figure 3:**
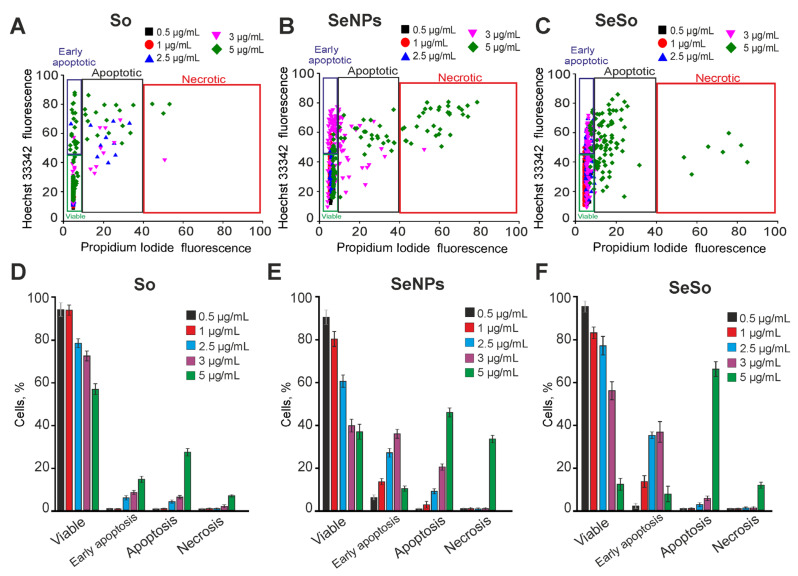
The effect of 48-h incubation of A-172 cells with various concentrations of sorafenib (So), selenium nanoparticles (SeNPs), and selenium-sorafenib nanocomplex (SeSo). (**A**–**C**)—The cytograms demonstrating the viability of A-172 cells after 48-h pre-incubation with different concentrations of So (**A**), SeNPs (**B**), and SeSo (**C**). X-axis: the intensity of PI fluorescence; Y-axis: the intensity of Hoechst 33342 fluorescence. Cells were stained with the probes after 48-h incubation with different concentrations of compounds. (**D**–**F**)—The comparison of the effect of different concentrations of So, SeNPs, and SeSo on A-172 cell survival after 48-h pre-incubation. Panels (**D**–**F**) is a calculation (technical replicate) of the data (mean ± SE) presented in panels (**A**–**C**), performed in four repetitions, *n* = 4. Cell images are shown in Supplementary Figures S4–S6.

An increase in the time of pre-incubation of A-172 cells with sorafenib up to 48 h led to a dose-dependent induction of the early stages of apoptosis in 10–18% of cells, starting from a concentration of 2.5 μg/mL. While the late stages of apoptosis were observed in 8–21% of cells, and single necrotic cells were observed only at an So concentration of 5 μg/mL (Figure 3A,D; Supplementary Figure S4). An increase in the time of incubation of A-172 cells with SeNPs up to 48 h led to an increase in the apoptotic effect of nanoparticles. At the same time, SeNPs at a concentration of 1–2.5 μg/mL induced early stages of apoptosis in 18–23% of cells with a trend towards an increase in the number of cells at the late stages of apoptosis (Figure 3B,E; Supplementary Figure S5). Incubation of cells with 3 μg/mL SeNPs for 48 h increased the number of cells at early and late stages of apoptosis up to 42% and 24%, respectively, while SeNPs at a concentration of 5 μg/mL induced apoptosis in 47% and necrosis in 36% of cells (Figure 3B,E; Supplementary Figure S5). Increasing the time of incubation of A-172 cells with SeSo to 48 h did not lead to a significant increase in the number of cells in the early stages of apoptosis, compared with 24 h of exposure. Using 1–3 μg/mL SeSo, early stages of apoptosis were registered in 18–37% of cells without induction of late stages of apoptosis or necrosis. Only at a high concentration of SeSo (5 μg/mL) did activation of the late stages of apoptosis occur in 78% of cells and necrosis in 16% of cells (Figure 3C,F; Supplementary Figure S6).

**Figure 4:**
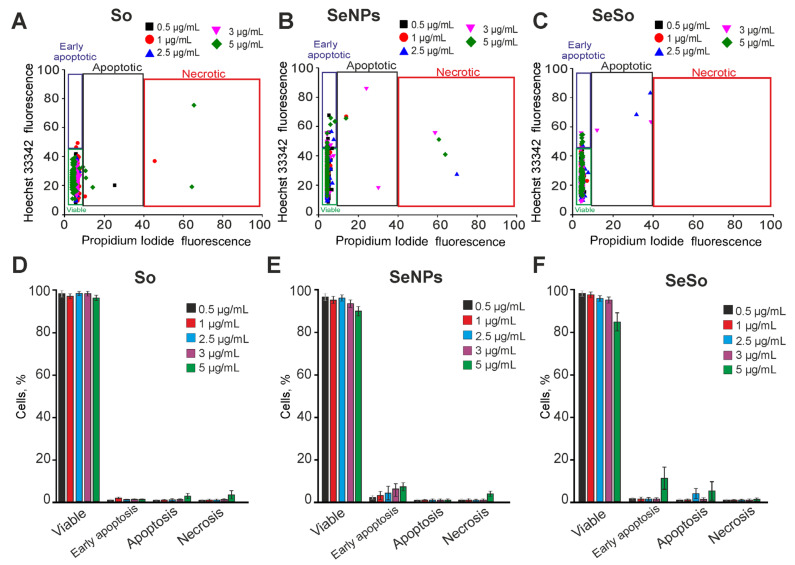
Effect of 24-h incubation of cortical astrocytes with various concentrations of sorafenib (So), selenium nanoparticles (SeNPs), and selenium-sorafenib nanocomplex (SeSo). (**A**–**C**)—Cytograms demonstrating the viability of astrocytes after 24-h pre-incubation with different concentrations of So (**A**), SeNPs (**B**), and SeSo (**C**). X-axis: the intensity of PI fluorescence; Y-axis: the intensity of Hoechst 33342 fluorescence. Cells were stained with the probes after 24-h incubation with different concentrations of compounds. (**D**–**F**) Comparison of the effect of different concentrations of So, SeNPs, and SeSo on the survival of astrocytes after 24-h pre-incubation. Panels (**D**–**F**) is a calculation (technical replicate) of the data (mean ± SE) presented in panels (**A**–**C**), performed in three repetitions, *n* = 3. Cell images are shown in Supplementary Figures S7–S9.

Mouse cortical astrocytes were cultured for 8 days, and then the test compounds were added to the culture medium for 24 h. The results of the vitality tests showed that 24-h incubation of astrocytes with So did not cause cell death, only with the addition of 5 μg/mL So, single cell necrosis was recorded (Figure 4A,D; Supplementary Figure S7). Pre-incubation of astrocytes with SeNPs for 24 h also did not cause cell death in the concentration range of 1–5 μg/mL; there was only a trend for a slight (up to 8–10%) increase in the number of cells at the early stage of apoptosis. Necrosis was registered only in single astrocytes at a SeNPs concentration of 5 μg/mL (Figure 4B,E; Supplementary Figure S8). The SeSo nanocomplex within 24 h after incubation did not lead to cell death. Only when cells were treated with 5 μg/mL SeSo, up to 10% of astrocytes were recorded at the early stage of apoptosis (Figure 4C,F; Supplementary Figure S9).

**Figure 5:**
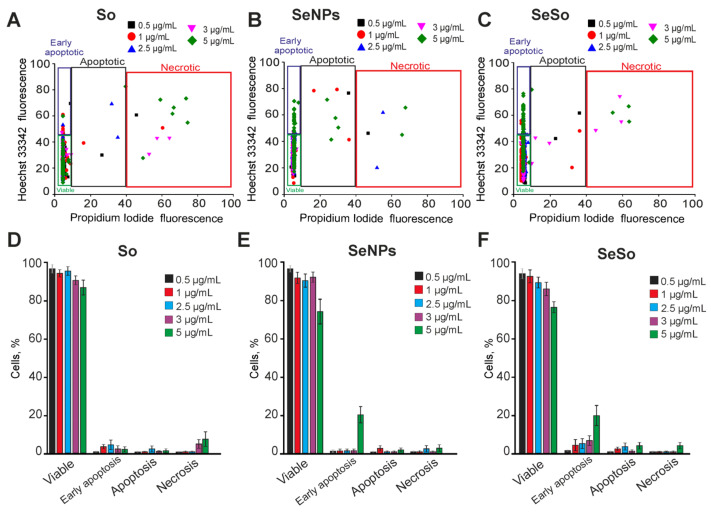
Effect of 48-h incubation of cortical astrocytes with various concentrations of sorafenib (So), selenium nanoparticles (SeNPs), and selenium-sorafenib nanocomplex (SeSo). (**A**–**C**)–Cytograms demonstrating the viability of astrocytes after 48-h pre-incubation with different concentrations of So (**A**), SeNPs (**B**), and SeSo (**C**). X-axis: the intensity of PI fluorescence; Y-axis: the intensity of Hoechst 33342 fluorescence. Cells were stained with the probes after 24-h incubation with different concentrations of compounds. (**D**–**F**) Comparison of the effect of different concentrations of So, SeNPs, and SeSo on survival of astrocytes after 48-h pre-incubation. Panels (**D**–**F**) is a calculation (technical replicate) of the data (mean ± SE) presented in panels (**A**–**C**), performed in three repetitions, *n* = 3. Cell images are shown in Supplementary Figures S10–S12.

An increase in the time of incubation of cortical astrocytes with So (from 0.5 μg/mL to 5 μg/mL) up to 48 h did not cause a significant induction of apoptosis—at early and late stages of apoptosis, no more than 10% of cells were registered. Using So concentrations of 3 μg/mL and 5 μg/mL induced necrosis in 6–11% of astrocytes (Figure 5A,D; Supplementary Figure S10). Incubation of astrocytes with SeNPs for 48 h also did not cause cell death, only a high concentration of nanoparticles—5 μg/mL—caused early stages of apoptosis in 22% of cells and necrosis in single astrocytes (Figure 5B,E; Supplementary Figure S11). Interestingly, 48 h after exposure of astrocytes with SeSo in the concentration range of 2.5–5 μg/mL induced activation of the early stages of apoptosis (Figure 5C,F; Supplementary Figure S12), which was not observed after 24 h pre-incubation (Figure 4).

Thus, sorafenib in the studied concentration range did not cause massive cell death activation of the A-172 cell line after 24 h exposure. Whereas, after 48 h, induction of early stages of apoptosis in 10–20% of cells and late stages of apoptosis in 10–30% of cells was observed after pre-incubation with high doses of sorafenib. Following a 24-h exposure to 2.5–5 μg/mL SeNPs, the appearance of cells with late stages of apoptosis was recorded. However, an increase in the incubation time of A-172 cells, even with low doses of SeNPs, contributed to the enhancement of apoptotic processes. The selenium-sorafenib nanocomplex in a wide range of concentrations induced the early stages of apoptosis in glioblastoma cells 24 h after application. Then, 48 h later, this effect increased, but without the induction of late stages of apoptosis (with the exception of 5 μg/mL). At the same time, in astrocytes of the cerebral cortex, sorafenib, selenium nanoparticles, and the selenium-sorafenib nanocomplex did not cause cell death either after 24 or after 48 h of incubation, which indicates the target orientation of the studied agents on cancer cells.

### 2.3. The Effect of Sorafenib, Selenium Nanoparticles, and the Selenium-Sorafenib Nanocomplex on the Expression of Genes Encoding ER Stress Proteins and Apoptosis Regulatory Proteins

**Figure 6:**
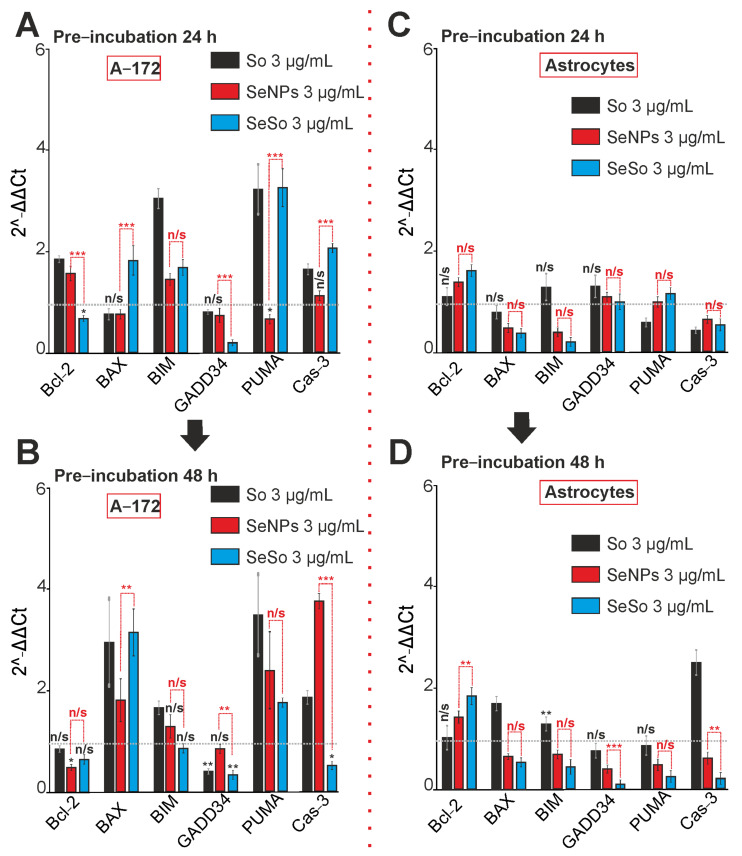
The expression patterns of genes encoding pro-apoptotic proteins in A-172 cells and cortical astrocytes after 24-h and 48-h incubation with 3 μg/mL of sorafenib (So), selenium nanoparticles (SeNPs), and selenium-sorafenib nanocomplex (SeSo). (**A**,**B**)—Gene expression in A-172 cells after 24 (**A**) and 48 h (**B**) pre-incubation with the studied agents. (**C**,**D**)—Gene expression in cortical astrocytes after 24 (**A**) and 48 h (**B**) pre-incubation with the studied agents. The level of gene expression in the control cells was taken as 1. Statistical significance was assessed using paired *t*-test. Comparison of experimental groups with control is indicated by black asterisks. Unlabeled columns—data significant vs. control, *** *p* < 0.001. n/s—data not significant (*p* > 0.05), * *p* < 0.05, ** *p* < 0.01 and *** *p* < 0.001. Significance comparisons between SeNPs and SeSo are indicated in red. *n* = 3.

The increase in [Ca^2+^]_i_ and the induction of apoptosis in glioblastoma cells under the action of SeNPs and SeSo occurred due to changes in the expression of genes that regulate cell death. To study the effects of So, SeNPs, and SeSo, A-172 human glioblastoma cells or astrocytes of the cerebral cortex were incubated with the studied compounds (3 μg/mL), and total RNA was isolated after 24 and 48 h. PCR analysis showed that after 24 and 48 h of incubation of glioblastoma cells with So, there was a significant increase in the expression of proapoptotic genes BIM, PUMA, and Cas-3 (Figure 6A,B). For cerebral cortex astrocytes, on the contrary, we detected a decrease in the expression of proapoptotic PUMA and Cas-3 genes after 24-h incubation with 3 μg/mL So (Figure 6C). At the same time, we did not observe a significant decrease in expression levels for other genes.

Treatment of astrocytes with SeNPs or SeSo for 24 h led to the suppression of the expression of proapoptotic BAX, BIM, and Cas-3, while the effect on the basic expression of apoptosis genes was more pronounced for SeSo (Figure 6C). Then, 48 h later, cell incubation with So also led to an increase in the expression levels of a number of proapoptotic genes in cerebral cortex astrocytes: BAX, BIM, and Cas-3 (Figure 6D). However, treatment of these cells with SeNPs and SeSo for 48 h still reduced the expression levels of all studied proapoptotic genes (Figure 6D).

**Figure 7:**
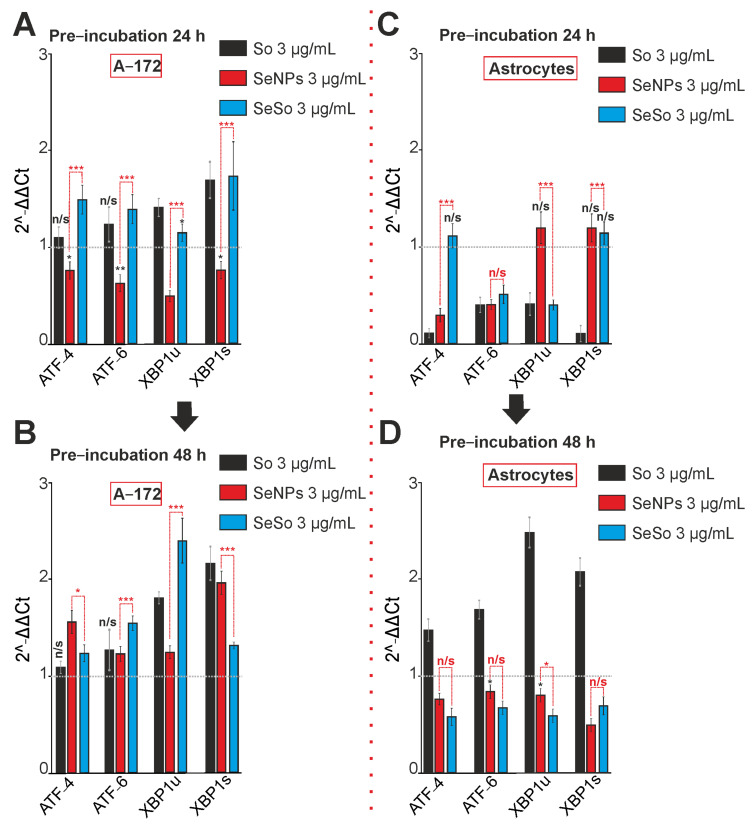
The expression patterns of genes encoding ER-stress proteins (**A**–**D**) involved in apoptosis activation in A-172 cells and cortical astrocytes after 24-h and 48-h incubation with 3 μg/mL of sorafenib (So), selenium nanoparticles (SeNPs), and selenium-sorafenib nanocomplex (SeSo). (**A**,**B**)—Gene expression in A-172 cells after 24 (**A**) and 48 h (**B**) pre-incubation with the studied agents. (**C**,**D**)—Gene expression in cortical astrocytes after 24 (**A**) and 48 h (**B**) pre-incubation with the studied agents. The level of gene expression in control cells was taken as 1. Statistical significance was assessed using paired *t*-test. Comparison of experimental groups with control is indicated by black asterisks. Unlabeled columns—data significant vs. control, *** *p* < 0.001. n/s—data not significant (*p* > 0.05), * *p* < 0.05, ** *p* < 0.01 and *** *p* < 0.001. Significance comparisons between SeNPs and SeSo are indicated in red. *n* = 3.

It is known that endoplasmic reticulum stress (ER-stress) is involved in the induction of apoptosis, on the one hand, but, on the other hand, it is involved in the activation of adaptive cytoprotective mechanisms. In our experiments, the treatment of A-172 glioblastoma cells for 24 and 48 h with sorafenib led to an increase in the expression of mRNA of the spliced form of the XBP1s transcription factor and did not change the expression of two other markers of ER-stress, ATF-4 and ATF-6 (Figure 7A,B). Treatment of cells with 3 μg/mL SeNPs for 24 h led to a significant decrease in the expression of all key ER-stress markers (Figure 7A), but after 48 h, on the contrary, a significant increase in their expression was observed (Figure 7B). Upon exposure of glioblastoma cells with SeSo for both 24 h (Figure 7A) and 48 h (Figure 7B), we observed a steady increase of genes encoding of ER-stress proteins.

At the same time, 24-h exposure to sorafenib of cortical astrocytes led to suppression of the expression of all the studied ER-stress genes (Figure 7C), and after 48 h, on the contrary, to an increase in their expression (Figure 7D). The application of SeNPs contributed to a decrease in ATF-4 and ATF-6 gene expression levels already after 24 h of exposure (Figure 7C), and after 48 h to a decrease in all three key markers of ER- stress (Figure 7D). Similarly, the SeSo nanocomplex reduced the level of two genes ATF-6 and XBP1u after 24 h (Figure 7C), and after 48 h of all genes, ATF-4, ATF-6, and XBP1s (Figure 7D).

**Figure 8:**
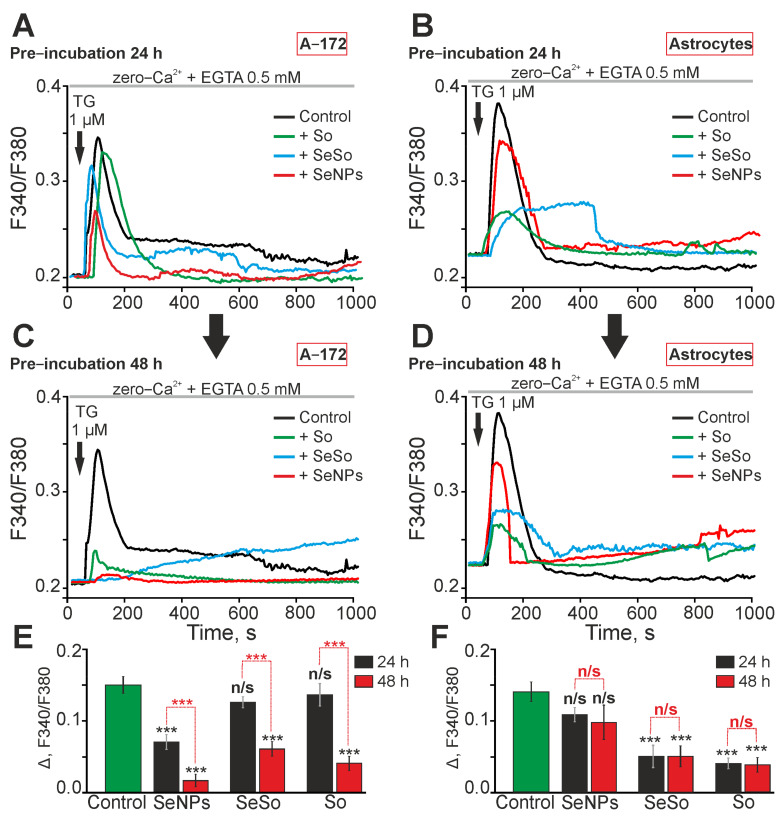
Effect of preincubation of A-172 human glioblastoma cells and astrocytes with So, SeSo, or SeNPs on ER Ca^2+^ capacity. (**A**,**C**,**E**)—Ca^2+^-responses of A-172 human glioblastoma cells in response to the application of thapsigargin (TG, 10 mkM) in a calcium-free medium supplemented with 0.5 mM EGTA after 24 (**A**) and 48 (**C**) h preincubation with 3 μg/mL So, SeSo, or SeNPs and their average amplitudes (**E**). (**B**,**D**,**F**) Ca^2+^-responses of mouse cerebral cortex astrocytes to the application of thapsigargin (TG, 10 mkM) in a calcium-free medium supplemented with 0.5 mM EGTA after 24 (**B**) and 48 (**D**) h of preincubation with 3 μg/mL So, SeSo, or SeNPs and their mean amplitudes (**F**). Ca^2+^-responses averaged over several tens of cells for panels (**A**–**D**) and their amplitudes (**D**,**F**) are shown. Statistical significance was assessed using paired *t*-test. Comparison of experimental groups with control is indicated by black asterisks. n/s—data not significant (*p* > 0.05) and *** *p* < 0.001. Significance comparisons between SeNPs and SeSo are indicated in red. The experiments were performed in three repetitions on three separate cell cultures.

ER-stress can lead to the disruption of cellular Ca^2+^ homeostasis and Ca^2+^ capacity of the endoplasmic reticulum [33,34]. Preincubation of A-172 cells with 3 μg/mL So or SeSo for 24 h did not lead to a significant decrease in the Ca^2+^ capacity of the ER, as evidenced by the amplitudes of Ca^2+^-responses in response to the addition of 10 μM thapsigargin in a nominally calcium-free medium (Figure 8A, green and blue curves; Figure 8E), comparable with the Ca^2+^ responses of control cells (Figure 8A, black curve; Figure 8E). At the same time, 24 h after incubation of cells with 3 μg/mL SeNPs, a significant suppression of Ca^2+^ responses to thapsigargin occurred, which indicates a decrease in the ER capacity for Ca^2+^ ions (Figure 8A,E). However, after a 48-h preincubation of the cells with the studied agents, a pronounced depletion of the ER occurred and thapsigargin evoked low-amplitude Ca^2+^-responses, and after exposure with SeSo, thapsigargin induced a slow increase in [Ca^2+^]_i_ (Figure 8C,E).

Incubation of astrocytes in the cerebral cortex for 24 h with 3 μg/mL SeNPs did not cause significant depletion of ER capacity, as evidenced by the generation of Ca^2+^-responses by cells in response to thapsigargin, comparable to the control (Figure 8B,F). At the same time, 24-h incubation of astrocytes with 3 μg/mL So or SeSo led to a decrease in the amplitude of the Ca^2+^-responses in response to the application of thapsigargin (Figure 8C,F), which indicates the depletion of the ER by these agents. Increasing the time of incubation of astrocytes with SeNPs, So, or SeSo to 48 h also did not lead to significant depletion of the ER, relative to 24 h of incubation with the studied agents (Figure 8D,F).

Thus, 24 h after incubation with A-172 glioblastoma cells, So and SeSo resulted in a moderate increase in genes encoding ER-stress proteins, which did not correlate with the depletion of ER Ca^2+^-stores. Whereas incubation with SeNPs, on the contrary, after 24 h caused suppression of the ER-stress genes expression and led to moderate depletion of the Ca^2+^ pool. At the same time, the level of proapoptotic genes increases in the case of incubation of glioblastoma cells with So and SeSo. An increase in the time of incubation of glioblastoma cells with the studied agents led to an increase in the level of expression of ER-stress genes and a significant depletion of the ER Ca^2+^ pool, which correlates with an increase in the expression of proapoptotic genes. At the same time, the most pronounced effect on the capacity of the ER Ca^2+^ pool, the expression of ER-stress and apoptosis genes was observed for “naked” SeNPs and the SeSo nanocomplex. For astrocytes of the cerebral cortex, we found that after 24 h of incubation, So and the SeSo nanocomplex lead to multidirectional effects on the expression of ER-stress genes, when So causes a decrease in expression, and SeSo causes an increase, but at the same time, a moderate depletion of the Ca^2+^ pool of the ER occurs when there is no effect or reduced expression of proapoptotic genes.

An increase in the time of incubation of astrocytes up to 48 h with SeNPs or SeSo led to a decrease in the expression of ER-stress genes without a significant depletion of the ER, which correlated with the suppression of apoptosis gene expression. On the contrary, after a 48-h action of So on astrocytes, there was an increase in the expression of ER-stress and apoptosis genes, without a significant depletion of the ER Ca^2+^ pool. Thus, it can be assumed that an increase in the expression of ER-stress genes leads to the most pronounced depletion of the ER Ca^2+^ pool and activation of apoptosis in glioblastoma cells, while in cortical astrocytes the effect of the studied agents on ER-stress and the induction of apoptosis is less pronounced after application of So or is opposite in the case of SeNPs or SeSo.

Thus, sorafenib has a weak effect on the gene expression of selenoproteins and selenium-containing proteins in glioblastoma cells after 24 and 48 h of exposure. The effect of SeNPs and the SeSo nanocomplex on the expression of these genes was observed only after 48 h. At the same time, in astrocytes of the cerebral cortex, the expression of selenoproteins and selenium-containing proteins is increased already after 24 and 48 h. It should be said that the action of SeNPs and SeSo has more pronounced effects on the expression of the studied genes in both cell types, compared with sorafenib.

### 2.4. The Effect of Sorafenib, Selenium Nanoparticles, and the Selenium-Sorafenib Nanocomplex on the Expression of Genes Encoding Selenium-Containing Proteins

**Figure 9:**
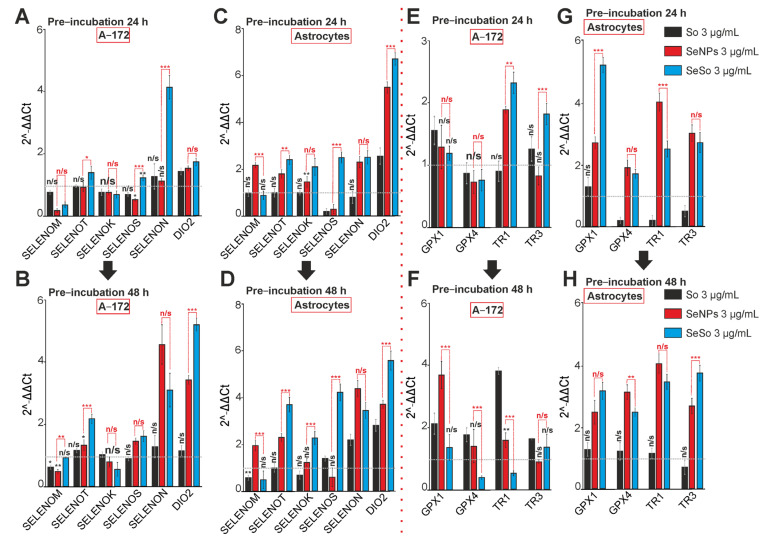
The expression patterns of genes encoding selenoproteins (**A**–**D**) and selenium-containing proteins (**E**–**H**) in A-172 cells and cortical astrocytes after 24-h and 48-h incubation with 3 μg/mL of sorafenib (So), selenium nanoparticles (SeNPs), and selenium-sorafenib nanocomplex (SeSo). The level of gene expression in control cells was taken as 1. Statistical significance was assessed using paired *t*-test. Comparison of experimental groups with control is indicated by black asterisks. Unlabeled columns—data significant vs. control, *** *p* < 0.001. n/s—data not significant (*p* > 0.05), * *p* < 0.05, ** *p* < 0.01 and *** *p* < 0.001. Significance comparisons between SeNPs and SeSo are indicated in red. *n* = 3.

Incubation of A-172 cells with 3 μg/mL So for 24 h does not significantly affect the expression of most selenoproteins and selenium-containing proteins. Of the 10 studied genes, only the DIO2 and GPX1 genes are up-regulated (Figure 9A,E). SeNPs, after 24 h lead to a decrease in the expression of SELENOM, SELENOS, and an increase in the level of DIO2 and TR2 (Figure 9A,E). Whereas SeSo inhibits the SELENOM expression after 24 h, but at the same time, the expression level of SELENOT, SELENOS, SELENON, DIO2, TR1, and TR3 increases (Figure 9A,E). 

Following 48 h of So exposure to A-172 glioblastoma cells, there was no significant change in the expression of genes encoding selenoproteins, and the level of genes encoding selenium-containing proteins GPX1, GPX4, TR1, and TR3 significantly increased (Figure 9B,F). Following a 48-h exposure to SeNPs, the trend towards a decrease in SELENOM expression persists, but there is an increase in the expression of SELENOT, SELENOS, SELENON, DIO2, GPX1, and TR1 (Figure 9B,F). Following the cell exposure with SeSo for 48 h, an even greater increase in the expression of SELENOT, SELENOS, SELENON, and DIO2 occurs, but occurs against the background of suppression of GPX4 and TR1 (Figure 9B,F). 

In astrocytes of the cerebral cortex, after a 24-h exposure, So leads to a decrease in the expression of SELENOS (Figure 9C) and GPX4, TR1, and TR3 (Figure 9G), while after 48 h, such exposure to So leads to the restoration of GPX4, TR1, and TR3 to control levels (Figure 9H), and the expression of selenoproteins SELENOS, SELENON, and DIO2 begins to increase against the background of suppression of SELENOM (Figure 9D). Selenium nanoparticles (SeNPs), after a 24-h exposure, lead to an increase in the expression of all selenoproteins, except for SELENOS (Figure 9C) and all studied selenium-containing proteins (Figure 9G), and the trend towards their growth persists after a 48-h exposure. exposure to SeNPs (Figure 9D,H). Both 24-h (Figure 9C,G) and 48-h (Figure 9D,H) exposure cortical astrocytes with SeSo lead to a similar or even more pronounced effect of enhancing the expression of genes encoding selenoproteins and selenium-containing proteins.

Thus, sorafenib has a weakly pronounced effect on the expression of selenoprotein and selenium-containing protein genes in A-172 glioblastoma cells after both 24 and 48-h incubation. The effect of SeNPs and the SeSo nanocomplex on the expression of these genes is observed after 48 h of incubation. At the same time, in the astrocytes of the cerebral cortex, there is an increase in the expression of selenoproteins and selenium-containing proteins already after 24 h and after 48 h, these effects are enhanced. It should be noted that the action of SeNPs and SeSo has more pronounced effects on the expression of the studied genes in both cell types, compared with sorafenib.

### 2.5. Effect of Sorafenib, Selenium Nanoparticles, and Selenium-Sorafenib Nanocomplex on the Expression of Genes Encoding Key Kinases

**Figure 10:**
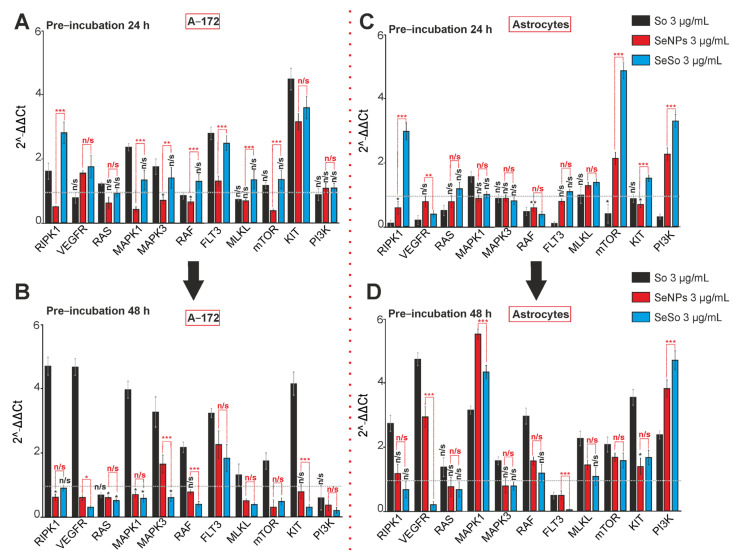
The expression patterns of genes encoding signal kinases in A-172 cells and cortical astrocytes after 24-h and 48-h incubation with 3 μg/mL of sorafenib (So), selenium nanoparticles (SeNPs), and selenium-sorafenib nanocomplex (SeSo). (**A**,**B)**—Gene expression in A-172 cell after 24-h (**A**) and 48-h (**B**) pre-incubation with So, SeNPs, and SeSo. (**C**,**D**)—Gene expression in cortical astrocytes after 24-h (**C**) and 48-h (**D**) pre-incubation with So, SeNPs, and SeSo. The level of gene expression in control cells was taken as 1. Statistical significance was assessed using paired *t*-test. Comparison of experimental groups with control is indicated by black asterisks. Unlabeled columns—data significant vs. control, *** *p* < 0.001. n/s—data not significant (*p* > 0.05), * *p* < 0.05, ** *p* < 0.01 and *** *p* < 0.001. Significance comparisons between SeNPs and SeSo are indicated in red. *n* = 3.

Protein kinases regulate almost all intracellular processes and, as a result, determine cell proliferation, differentiation, survival, and death of both normal and cancer cells [35,36]. Following a 24-h incubation of glioblastoma cells with 3 μg/mL So, either there was no change in the expression of genes encoding kinases (VEGFR, RAS,RAF, MLKL, mTOR, and PI3K), or an increase in their expression was observed—RIPK1, MAPK1, MAPK3, FLT3, and KIT (Figure 10A—black columns). A similar effect was exerted on cells by the 24-h SeSo nanocomplex (Figure 10A, blue columns), but the increase in gene expression was less pronounced, compared to So.

However, already after 48 h of exposure of A-172 cells to sorafenib, a strong increase in the expression of most of the studied kinase genes was observed, with the exception of RAS, MLKL, and PI3K (Figure 10B, black bars). However, in the case of the SeSo nanocomplex, after 48 h, on the contrary, a pronounced inhibition of the expression of the overwhelming majority of the studied kinases was observed (Figure 10B, blue columns), with the exception of RIPK1 and FLT3, the expression level of which did not change. Following 24 h of incubation, selenium nanoparticles led to a pronounced increase in the expression of only the VEGFR and KIT genes (Figure 10A, red columns). Following 48 h of incubation of A-172 cells with SeNPs, suppression of the expression of most kinase-encoding genes, comparable with SeSo, was observed, with the exception of MAPK3 and FLT3, the level of which increased, and RAF and KIT, the expression of which did not change (Figure 10B, red columns).

In cortical astrocytes, the opposite effect was observed, relative to A-172 glioblastoma. In astrocytes, after a 24-h incubation with 3 μg/mL So, the expression of most protein kinases was inhibited, with the exception of MAPK1 (Figure 10C, black bars). Whereas after 48 h the situation with the gene expression changed cardinally, there was a pronounced increase in the expression of all kinases, except for FLT3 (Figure 10D, black columns).

Following a 24-h incubation of astrocytes with SeNPs, there was a significant increase in the expression of MLKL, mTOR, and PI3K and a decrease in RIPK1, RAF, and KIT (Figure 10C, red columns). Following 48 h of incubation with SeNPs, there was a sharp increase in the expression of VEGFR, MAPK1, RAF, MLKL, mTOR, KIT, and PI3K, and the level of FLT3 decreased (Figure 10D, red columns). The SeSo nanocomplex, after a 24-h exposure to astrocytes, led to an increase in the expression of the RIPK1, RAS, MLKL, mTOR, and PI3K genes, while a decrease in the expression of VEGFR and RAF was observed (Figure 10C, blue columns). However, after 48 h of incubation, the expression of most of these genes decreased to the control level or below (Figure 10D, blue columns). However, in this case a high level of genes expression was registered for the MAPK1, mTOR, KIT, and PI3K genes (Figure 10D, blue columns).

Thus, sorafenib at the studied concentration, not only did not suppress the expression of genes encoding kinases, but even led to its increase, especially after 48 h of incubation, which may indicate the resistance of A-172 glioblastoma cells to the action of this dose of So. Incubation of selenium nanoparticles with A-172 cells for 24 h, such as sorafenib, led to the suppression of the kinase expression, and this effect increased after 48 h. The SeSo nanocomplex had effects similar to the use of sorafenib, i.e., after 24 h of treatment, the expression of kinase genes increased; however, after 48 h, the effect changed dramatically and the expression of most kinases was suppressed, comparable to the effect of “naked” SeNPs. In cortical astrocytes after 24 h, noticeable effects on the expression of kinases were registered in the case of sorafenib, which generally suppressed the expression of most kinases. However, after 48 h of incubation, all tested agents led to the increased expression of most kinases.

## 3. Discussion

We have previously shown that the application of selenium nanoparticles, as well as the selenium and sorafenib nanocomplex, caused the generation of Ca^2+^ responses in HepG2 hepatocellular carcinoma cells. At the same time, the nanocomplex caused the generation of Ca^2+^-responses in cells at a concentration three times lower than that of SeNPs [32]. We have shown here that the Ca^2+^ signaling system of A-172 glioblastoma cells and cortical astrocytes is more sensitive to the action of naked selenium nanoparticles, compared to the nanocomplex. At the same time, there is some evidence that preincubation of cardiomyocytes with high concentrations (10–30 μM) of sorafenib causes the release of Ca^2+^ ions from the sarcoplasmic reticulum and its depletion by more than 60%. This action of sorafenib suggests one of the pathways through which it can disrupt the functioning of the cardiovascular system by suppressing contraction [37]. In our experiments, acute application of sorafenib in the concentration range of 1–10 μg/mL to A-172 cancer cells or cerebral cortex astrocytes did not cause an increase in [Ca^2+^]_i_, in contrast to the SeSo nanocomplex. However, long-term incubation of glioblastoma cells with all tested agents resulted in depletion of ER stores for calcium after 48 h. In astrocytes, this effect was observed when treated with sorafenib after 24 h, but was of a moderate nature.

It is known that Ca^2+^ ions as a second messenger regulate almost all cellular functions, including proliferation. There is strong evidence of calcium-dependent suppression of proliferation and migration of cancer cells by sorafenib, which occurs due to the deactivation of FAK (focal adhesion kinase) and inhibition of Akt signaling. For example, the addition of lactate calcium salt to colorectal cancer cells causes an increase in [Ca^2+^]_i,_ which correlates with the inhibition of the above signaling pathways [38]. A continuous influx of Ca^2+^ ions into the cytosol is one of the mechanisms of apoptosis activation due to calcium-dependent activation of proteolytic and catabolic degradation enzymes (proteases and phosphatases) [39]. There is convincing evidence of the low sensitivity of glioblastoma and glioma cells to sorafenib therapy. It has been shown that in the T98G glioblastoma cell line, sorafenib at concentrations of 0–5 μM, does not induce significant cell death [31]. Similarly, sorafenib did not induce apoptosis after 24 h of incubation. Only after 48 h did the proportion of cells appear in the early stages of apoptosis after pre-incubation with high doses of So. On the contrary, SeNPs and SeSo already after 24 h led to the activation of predominantly early stages of apoptosis in A-172 glioblastoma cells, and this effect increased, turning into late apoptosis, after 48 h of incubation. Other lines of glioblastoma cells showed proapoptotic effects of sorafenib only after 48 h of exposure. U373 and U87 glioblastoma cells were treated with various concentrations of sorafenib, and only 48 h later apoptosis induction was recorded in the concentration range ≥5 μg/mL. Whereas in our experiments, the induction of the early stages of apoptosis was recorded already at a SeSo nanocomplex concentration of 1 μg/mL. For sorafenib, an increase in the proapoptotic effect was also shown under the action of tumor-treating fields (electric fields with alternating low and intermediate intensity), when after 48 h there was a significant increase in the number of cells in the early stages of apoptosis [40], which we also observed under the action of sorafenib, as part of a nanocomplex with selenium.

Thus, nanoselenium and sorafenib, as part of a nanocomplex with selenium, have a more pronounced proapoptotic effect on glioblastoma cells, even at low concentrations. At the same time, in LN-18 glioma cells of the line, sorafenib induced apoptosis in 39% of cells at a concentration of 1 μM, while at concentrations of 0.25, 0.5, 0.75, and 5 μM, the apoptotic effect was less pronounced. Moreover, the concentration of sorafenib 5 μM caused predominantly cell necrosis, i.e., injury rather than programmed cell death was initiated, which indicates a negative effect of this dose [41]. In our experiments, the SeNPs we developed at a concentration 5 μg/mL after 48 h could also lead to necrotic death of a small part of A-172 glioblastoma cells, and doping of these nanoparticles with sorafenib suppressed the induction of necrosis, which indicates an improvement in the quality of nanoparticles. There is strong evidence that sorafenib induces apoptosis predominantly in cancer cells, but significant cell death can be induced in non-cancer cells through the robust expression of p53-upregulated-modulator-of-apoptosis (PUMA) [42]. Our experiments show that both free sorafenib and the SeSo nanocomplex did not cause cell death in cortical astrocytes.

Activated astrocytes and microglia release pro-inflammatory cytokines (COX-2, IL-1β, IL-6, iNOS) into the brain tissue, which can lead to neuroinflammation [43]. The effects of sorafenib on neuroinflammation are shown in in vitro and in vivo models. Sorafenib suppresses the expression of mRNA of the pro-inflammatory cytokines COX-2 and IL-1β in microglial cells (BV2 cell line) under the action of liposaccharide. In addition, 5xFAD mice (an Alzheimer’s disease model) showed that sorafenib administered daily for 3 days resulted in the suppression of astrogliosis, but not microgliosis [44]. As for the nanoselenium cassette, we have previously shown the effect of activation of reactive A2-type astrogliosis, which activates the defense mechanisms of brain cortex cells and promotes the survival of neuroglial cultures under conditions of ischemia [20]. In response to infection or inflammation, astrocyte reactivation occurs, leading to the release of pro-inflammatory cytokines, changes in the morphology of astrocytes and their migration [45]. Sorafenib has been shown to suppress reactive astrogliosis in the cerebral cortex under toxic effects of LPS, which may be a promising strategy for the treatment of neuroinflammation [44]. In our experiments, after exposure to SeNPs and especially SeSo, the expression of genes encoding proapoptotic proteins in astrocytes decreased, which may contribute to the survival of astrocytes under damaging factors of various nature. While in glioblastoma cells, the same exposure to SeNPs or SeSo, on the contrary, increased the expression of apoptotic proteins. Dynamics of [Ca^2+^]_i_, ER stress, and cell death induction are closely related. On the one hand, ER stress, which plays a key role in tumor pathogenesis, can lead to tumor progression and resistance to chemoradiotherapy [46,47]. On the other hand, prolonged ER stress damages the biological function of cells and mediated cell apoptosis [48,49]. It has been shown that sorafenib can enhance endoplasmic reticulum (ER) stress-mediated apoptosis, and ER stress and unfolded protein response are also the mechanisms by which cancer cells resist drug therapy [50]. However, sorafenib in the nanocomplex with selenium, on the contrary, contributed to the induction of apoptosis. GRP78 is a chief regulator of ER function, and PERK, IRE1, and ATF6 were activated while GRP78 was released from these complexes, resulting in the induction of apoptosis [50,51]. In our experiments, SeNPs and SeSo led to the increased expression of key ER stress genes in glioblastoma cells, which correlated with the increased expression of proapoptotic genes as well. The effect of SeNPs and SeSo increased after 48 h of incubation with cells, which correlated with the depletion of the Ca^2+^ pool of the endoplasmic reticulum. In cortical astrocytes, on the contrary, there was a decrease in the expression of ER stress genes with a moderate depletion of the Ca^2+^ pool, which can be considered adaptive ER stress, since the activation of antiapoptotic proteins was also recorded and apoptosis was not induced.

Selenoproteins localized in the ER are involved in processes occurring in the ER, the most common of which are participation in protein degradation, regulation of ER stress, and redox metabolism. In our experiments, under the action of SeNPs and SeSo, SELENOM expression was suppressed, which may contribute to the induction of apoptosis. In cortical astrocytes, on the contrary, such influences promoted the expression of this selenoprotein. It has been shown that SELENOM regulates the activity of antioxidant enzymes in different tissues in different ways. In addition, the overexpression of SELENOM in HT22 and C8-D1A cells increases the concentration of cytosolic calcium in response to oxidative stress and may be involved in the regulation of apoptosis, blocking or delaying it [52]. SELENOT is similar in structure to SELENOM. Interestingly, in astrocytes of the cerebral cortex, the effect of SeNPs and SeSo promoted an increase in SELENOT expression, which can be regarded as a cytoprotective effect. It is known that SELENOT has a neuroprotective effect and promotes the survival of dopaminergic neurons and inhibits oxidative stress [53]. In A-172 cancer cells, no effect on SELENOT expression under the action of the investigated agents was recorded. Other authors have shown that a decrease in the expression of the SELENOS gene under conditions of ER stress led to an increase in Tau protein phosphorylation and a decrease in cell viability [54].

In our experiments, the most pronounced increase in SELENOS expression occurred in cortical astrocytes and under the action of the SeSo nanocomplex, while in glioblastoma cells, this effect of increased expression was less pronounced after 48-h incubation with SeSo. SELENON can play an important role in protecting cells from oxidative stress and maintaining Ca^2+^ homeostasis [55,56]. Therefore, SELENON deficiency increases the sensitivity of cells to the consequences of ER stress, increases the lipotoxicity of saturated fatty acids, while reducing insulin sensitivity [57]. In our experiments, all studied agents led to an increase in SELENON expression, but the SeSo nanocomplex turned out to be especially effective, which in the case of astrocytes can be regarded as a positive effect aimed at enhancing their viability. DIO2 is localized in the ER, unlike other types of deiodinases. Suppression of DIO2 led to an increase in ROS, a serious impairment of mitochondrial respiration, significant fragmentation of mitochondria, and a decrease in cell viability [58]. Pre-incubation with SeNPs and SeSo resulted in an increase in the expression of the DIO2 and SELENON genes in both cancer cells and cortical astrocytes, which improved the viability of cortical astrocytes. Whereas for A-172 glioblastoma cells, an increase in the expression of these proteins can be the cause of compensatory mechanisms observed against the apoptosis induction and cell death.

Increased expression of glutathione reductases and thioredoxin peroxidases after exposure to SeNPs and SeSo can be considered an activation of the antioxidant systems of astrocytes in the cerebral cortex. Following a 48-h exposure glioblastoma cells with SeSo, suppression of the gene expression encoding these proteins was observed. This changes can contribute to the suppression of the redox status and death of cancer cells. It has been shown that selenium-containing proteins, glutathione peroxidase and thioredoxin reductase, can counteract oxidative stress through reductive enzymatic activity [59,60].

There is evidence that VEGFRs are expressed at an increased level in glioblastoma cells and astrocytic tumor cells, and PDGFRb (tyrosine 75) is characterized by increased phosphorylation, compared to normal astrocytes [61,62]. In our experiments, the treatment of glioblastoma cells with SeNPs and SeSo led after 48 h to a decrease in the expression of these kinases, as well as most other kinases involved in oncogenesis. In the astrocytes of the cerebral cortex, on the contrary, either there was no effect of these agents, or there was an increase in expression, primarily of the cytoprotective PI3K. AMPK/mTOR signaling pathway activation is involved in tumorigenesis [63]. In our experiments, SeNPs and SeSo proved to be the most effective in suppressing the mTOR expression in glioblastoma cells, compared to the same concentration of So. In astrocytes, on the contrary, under the action of SeNPs and SeSo, mTOR expression increased, which indicates an increase in cell survival pathways [64]. It assumed that the use of the SeSo nanocomplex for the treatment of glioblastomas can lead to the suppression of oncogenicity of cancer cells and, at the same time, promote the survival of a healthy microenvironment. At the same time, PI3K is a kinase responsible for cell survival [65,66]. Following 48 h of pre-incubation with SeNPs or SeSo the expression of this kinase in glioblastoma cells was suppressed and correlates with the activation of apoptosis. Whereas in astrocytes of the cerebral cortex, on the contrary, these agents caused a significant increase in the PI3K expression and apoptosis was practically not recorded.

## 4. Materials and Methods

### 4.1. Reagents

From Sigma-Aldrich, St. Louis, USA: Adenosine 5′-triphosphatedisodiumsalthydrate (ATP, A1852), DMEM (D6429), Hanks′ balanced salt solution (HBSS, H4641), HEPES sodium salt (H7006); from Molecularprobes, Eugene, OR, USA: Hoechst 33342 (H1399), Propidium iodide (P1304MP); from Evrogene, Moscow, Russia: MMLV reverse transcriptase (SK022S), SYBR Green I PCR Master Mix (PK147L); from Thermo Fisher Scientific, Waltham, MA, USA: Fura-2AM (Cat. #F1221), fetal bovine serum (10099141); Selenum nanoparticles and sorafenib-doped selenium nanoparticles (SeSo) (courtesy of Dr. SV Gudkov, Prokhorov General Physics Institute, Russian Academy of Sciences, Moscow, Russia).

### 4.2. Preparation of Selenium Nanoparticles and Sorafenib-Doped Selenium Nanoparticles (SeSo)

**Figure 11:**
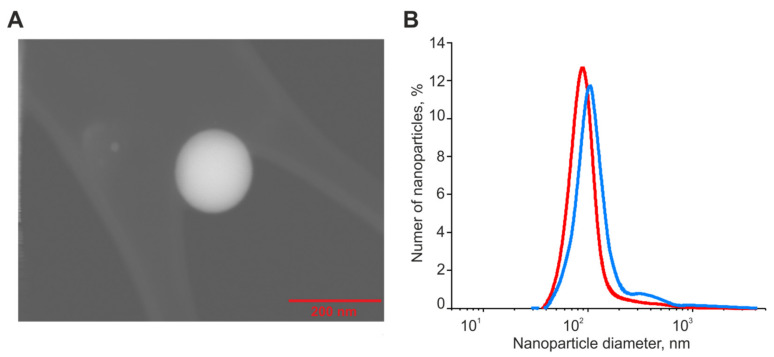
Size of SeNPs and SeSo nanoparticles. (**A**)—SeSo nanoparticle size in dry form obtained using a Carl Zeiss Libra 200FE transmission electron microscope. (**B**)—Hydrodynamic diameter of SeNPs and SeSo measured with dynamic light scattering Malvern Zetasizer Ultra Red Label 10 using MADLS (multi angle dynamic light scattering) technology. PDI_SeNPS_ = 0.22 ± 0.3, PDI_SeSo_ = 0.31 ± 0.5.

Selenium nanoparticles were obtained by laser ablation in deionized water with a resistivity of 18 MΩcm (Figure 11A). Briefly, a solid target was placed at the bottom of the cuvette under a thin layer of water (no thicker than 2–3 mm). A solid target was irradiated with a laser beam through a thin layer of water (λ = 1064 nm; T = 4–200 ns; f = 20 kHz; P = 20 W; E_p_ = 1 mJ). The mixing of the laser beam on the target was carried out along a given trajectory in the form of parallel straight lines inscribed in a rectangle using an LScanH galvanomechanical scanner (Ateko-TM, Russia). The average size of the nanoparticles is about 100 nm, the half-width is in the range of 70–130 nm. The zeta potential of the nanoparticles is about -30 mV. Sorafenib (Bayer HealthCare AG, Leverkusen, Germany) was applied to selenium nanoparticles as follows. A solution of sorafenib was prepared in citrate buffer (pH 4.1). Selenium nanoparticles were added to the sorafenib solution and incubated for 30 min. The amount of sorafenib associated with nanoparticles was determined by the change in the absorption spectrum of the solution before the addition of nanoparticles and after the deposition of nanoparticles. The concentration of sorafenib colloidal solution is 30 mg/mL. The concentration of nanoparticles is 10^11^ mL^−1^. The citrate buffer had a molarity of 0.1 M. The average particle size was measured using dynamic light scattering (DLS) and transmission electron microscopy (TEM) methods. We used a DLS Malvern Zetasizer Ultra Red Label 10 with MODIS (multi angle dynamic light scattering) technology and TEM Carl Zeiss Libra 200FE. The zeta potential was measured using Malvern Zetasizer Ultra Red Label 10. Information about the polydispersity index is included in the figure captions with DLS results (PDI_SeNPs_ = 0.22 ± 0.3, PDI_SeSo_ = 0.31 ± 0.5) (Figure 11B). The nanoparticles were purified from unloaded drug by centrifugation. Following each centrifugation, the SeSo nanoparticles were dissolved in sorafenib-free water and centrifuged again. We believe that sorafenib in the free form can only be present in trace amounts. Following purification by centrifugation, the supernatant does not have the characteristic multimode absorption spectrum of sorafenib. As for the second part of the commentary, we believe that 10^11^ mL^−1^ nanoparticles contain 12.07 mg/mL on their surface. The method of preparation, doping, and characterization of the obtained nanoparticles and nanocomplexes are described in detail in our previous work [32].

### 4.3. Cell Culture

A-172 cells (Human brain glioblastoma cell line) were purchased from ATCC (Manassas, VA, USA) and were grown on round coverslips for 48 h in a CO_2_-incubator in DMEM medium supplemented with 10% fetal bovine serum until a confluence of 80–95% was achieved. Cell cultures were used from the third passage. We checked our continuous cell culture for mycoplasma contamination every three months by PCR, as recommended by Drexler and Uphoff [67]. No mycoplasma contamination was detected.

Primary astroglial cell cultures were prepared from the brain cortex of mouse pups (P2–P3), as described [68]. The animals were euthanized by isoflurane overdose, the brains were removed, and the cortex was dissected out. Cortical tissue cuts from one animal were used for each cell culture preparation. Following isolation, the cells were plated on poly-d-lysine-coated glass coverslips and maintained at 37 °C in a humidified atmosphere of 5% CO_2_ and 95% air. Experiments were performed after 8 days of cultivation.

### 4.4. Assessment of the Cell Viability and Apoptosis

Cell death (apoptosis or necrosis processes) in the cell culture was assessed by simultaneous staining of cells with propidium iodide (PI, 1 μM) and Hoechst 33342 (HO342, 1 μM). Viable cells are not permeable to PI, while Hoechst 33342 penetrates through the plasma membrane and staining the chromatin. According to a commonly used method [69,70], cells were defined as apoptotic if the intensity of Hoechst 33342 fluorescence was 3–4 times higher, compared to Hoechst 33342 fluorescence in healthy cells, indicating chromatin condensation, which can occur as a result of apoptosis induction. The fluorescence of the probes was registered with a fluorescent system based on an inverted fluorescent microscope Axio Observer Z1 equipped with a high-speed monochrome CCD-camera Hamamatsu ORCA-Flash 2.8. The Lambda DG-4 Plus illuminator (Sutter Instruments, USA) was used as a source of excitation. To excite and record fluorescence of the probes we used: Filter Set 01 with excitation filter BP 365/12, beam splitter FT395, emission filter LP 397; Filter Set 20 with excitation filter BP 546/12, beam splitter FT560, emission filter BP 575–640. We used objective HCX PL APO 20.0 × 0.70 IMM UV, refraction index 1.52. Camera settings were500 pixels × 500 pixels (Voxel Size 0.724 μm × 0.723 μm), binning 2 × 2, resolution 14 bits. Five different fields of view were analyzed for each coverslip with cells. There were 100 or more cells in each field of view of the microscope. Each experiment was repeated three times with separate cell cultures. This approach allows analyzing several hundreds of cells in one experiment, and with repetitions it allows to obtain reliable quantitative and qualitative results, comparable to flow cytometry.

### 4.5. Fluorescent Ca^2+^ Measurements

Experiments were carried out in the daytime. The measurements of [Ca^2+^]_i_ were performed by fluorescence microscopy using Fura-2/AM. A-172 cells were loaded with the probe dissolved in Hanks balanced salt solution (HBSS) composed of (mM): 156 NaCl, 3 KCl, 2MgSO_4_, 1.25 KH_2_PO_4_, 2CaCl_2_, 10 glucose, and 10 HEPES, pH 7.4 at 37 °C for 40 min with subsequent 15 min washout. To measure the free cytosolic Ca^2+^ concentration, we used the Carl Zeiss Cell Observer and an inverted motorized microscope Axiovert 200M with a high-speed monochrome CCD-camera AxioCam HSm with a high-speed light filter replacing system, Ludl MAC5000. Fura-2 excitation and registration was recorded, using a 21HE filter set (Carl Zeiss, Oberkochen, Germany) with excitation filters BP340/30 and BP387/15, beam splitter FT-409 and emission filter BP510/90, objective lens HC PL Fluotar 10×/0.3 Dry, refraction index 1, excitation light source HBO 103W/2. Camera settings were 500 pixels × 500 pixels (Voxel Size 0.724 μm × 0.723 μm), binning 2 × 2, resolution 14 bits [17]. Background fluorescence was subtracted frame by frame using the Math Subtract plugin in ImageJ. Calcium responses were shown as a ratio of Fura-2 fluorescence intensities. Therefore we determined the amplitudes of Ca^2+^ responses to SeNPs as (Δ)—Fmax–Fmin of Fura-2 fluorescence and an increase in Fura-2 fluorescence reflects a linear [Ca^2+^]_i_ increase in response to receptor agonists. ImageJ 2002 software (RRID: SCR_003070) was used to analyze data. 

### 4.6. Extraction of RNA and Real-Time Polymerase Chain Reaction (RT-qPCR)

Total RNA from A-172 cells and cortical astrocytes after 24 h or 48 h of treatment with various concentrations of SeNP or SeSo were isolated using ExtractRNA reagent, according to the manufacturer’s instructions (Evrogene, Moscow, Russia). The concentration and purity of the total RNA were determined spectrophotometrically at 260/280 nm. First-strand cDNA was synthesized from 1–2 μg of total RNA using MMLV reverse transcriptase, according to the manufacturer’s instructions (Evrogene, Moscow, Russia). Real-time qPCRs were performed in a 25μL reaction mixture containing SYBR Green I PCR Master Mix (Evrogene, Russia) and 300 nM of the appropriate primers. The PCR procedure consisted of 94 °C for 2 min followed by 35 cycles of 94 °C for 1 min, 60 °C for 30 s, and 72 °C for 30 s. Glyceraldehyde-3-phosphate dehydrogenase (GAPDH) was used as an internal control for normalization, and results were expressed as 2-∆(∆∆Ct).

### 4.7. Statistical Analysis

All presented data were obtained from at least three cell cultures from two or three different passages. All values are given as mean ± standard error (SEM) or typical Ca^2+^-responses. Statistical analyses were performed by paired *t*-test. ImageJ, Origin 2016 (OriginLab, Northampton, MA, USA), and Prism GraphPad 7 (GraphPad Software Inc., CA, San Diego, USA, RRID: SCR_002798) software was used for data and statistical analysis. 

## 5. Conclusions

A-172 Human glioblastoma cells are highly resistant to the action of sorafenib, since its pro-apoptotic effect is recorded only after 48 h of exposure and at high doses. Selenium nanoparticles (SeNPs) and selenium-sorafenib nanocomplex (SeSo), in contrast to sorafenib (So), induce apoptosis in glioblastoma cells already after 24 h of exposure and at significantly lower concentrations. SeNPs and SeSo activate the Ca^2+^ signaling system of glioblastoma cells, cause ER stress, and induce apoptosis through regulation of the expression of selenoproteins and a number of protein kinases. At the same time, SeNPs and SeSo also activate the Ca^2+^ signaling system of cortical astrocytes. In this case, adaptive ER- stress occurs, which contributes to a change in genome expression aimed to increase the survival of these cells. According to a number of parameters, the SeSo nanocomplex turned out to be more effective than “naked” SeNPs as an anticancer agent in the case of glioblastoma cells and as a cytoprotector in the case of cortical astrocytes.

## Data Availability

The data presented in this study are available upon request from the corresponding author.

## Supplementary Materials

The following supporting information can be downloaded at: https://www.mdpi.com/article/10.3390/ijms24032411/s1 Figure S1: Induction of necrosis and apoptosis in the A-172 cells after 24 h incubation with various 0.5, 1, 2.5, 3 and 5 μg/mL concentrations of sorafenib (So). Figure S2: Induction of necrosis and apoptosis in the A-172 cells after 24 h incubation with various 0.5, 1, 2.5, 3 and 5 μg/mL concentrations of 50 nm selenium nanoparticles (SeNPs). Figure S3: Induction of necrosis and apoptosis in the A-172 cells after 24 h incubation with various 0.5, 1, 2.5, 3 and 5 μg/mL concentrations of 50 nm selenium nanoparticles doped with sorafenib (SeSo). Figure S4: Induction of necrosis and apoptosis in the A-172 cells after 48 h incubation with various 0.5, 1, 2.5, 3 and 5 μg/mL concentrations of sorafenib (So). Figure S5: Induction of necrosis and apoptosis in the A-172 cells after 48 h incubation with various 0.5, 1, 2.5, 3 and 5 μg/mL concentrations of 50 nm selenium nanoparticles (SeNPs). Figure S6: Induction of necrosis and apoptosis in the A-172 cells after 48 h incubation with various 0.5, 1, 2.5, 3 and 5 μg/mL concentrations of 50 nm selenium nanoparticles doped with sorafenib (SeSo). Figure S7: Induction of necrosis and apoptosis in the cortical astrocytes after 24 h incubation with various 0.5, 1, 2.5, 3 and 5 μg/mL concentrations of sorafenib (So). Figure S8: Induction of necrosis and apoptosis in the cortical astrocytes after 24 h incubation with various 0.5, 1, 2.5, 3 and 5 μg/mL concentrations of 50 nm selenium nanoparticles (SeNPs). Figure S9: Induction of necrosis and apoptosis in the cortical astrocytes after 24 h incubation with various 0.5, 1, 2.5, 3 and 5 μg/mL concentrations of 50 nm selenium nanoparticles doped with sorafenib (SeSo). Figure S10: Induction of necrosis and apoptosis in the cortical astrocytes after 48 h incubation with various 0.5, 1, 2.5, 3 and 5 μg/mL concentrations of sorafenib (So). Figure S11: Induction of necrosis and apoptosis in the cortical astrocytes after 48 h incubation with various 0.5, 1, 2.5, 3 and 5 μg/mL concentrations of 50 nm selenium nanoparticles (SeNPs). Figure S12: Induction of necrosis and apoptosis in the cortical astrocytes after 48 h incubation with various 0.5, 1, 2.5, 3 and 5 μg/mL concentrations of 50 nm selenium nanoparticles doped with sorafenib (SeSo).
