# Supplementary material for: Ca2+-Dependent Effects of the Selenium-Sorafenib Nanocomplex on Glioblastoma Cells and Astrocytes of the Cerebral Cortex: Anticancer Agent and Cytoprotector
Source: Int J Mol Sci. 2023 Jan 26;24(3):2411. doi: 10.3390/ijms24032411 (PMC9917080; doi:10.3390/ijms24032411)
Supplement: Supplementary file 1 [file ijms-24-02411-s001.zip › ijms-2107705-supplementary.pdf]

A-172, 24 h incubation with So

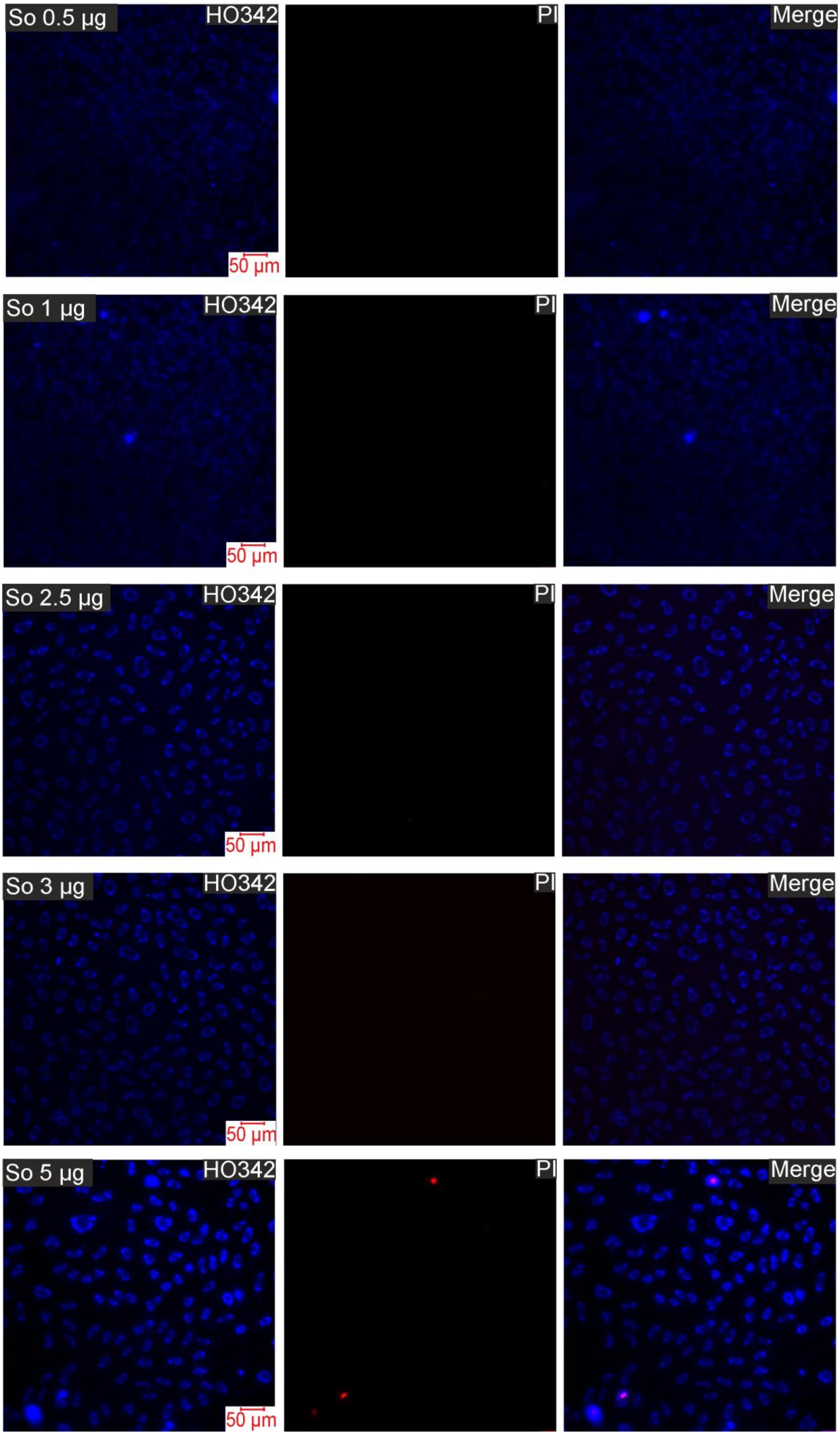

SUPPLEMENTARY, FIGURE S1. Induction of necrosis and apoptosis in the A-172 cells after 24 hour incubation with various 0.5, 1, 2.5, 3 and 5 μg/mL concentrations of sorafenib (So). Double staining of cells with Hoechst 33342 (HO342), Propidium iodide (PI) and merge (Merge). The images shown in the figure correspond to the data in figure 2 of the text of the manuscript.

A-172, 24 h incubation with SeNPs

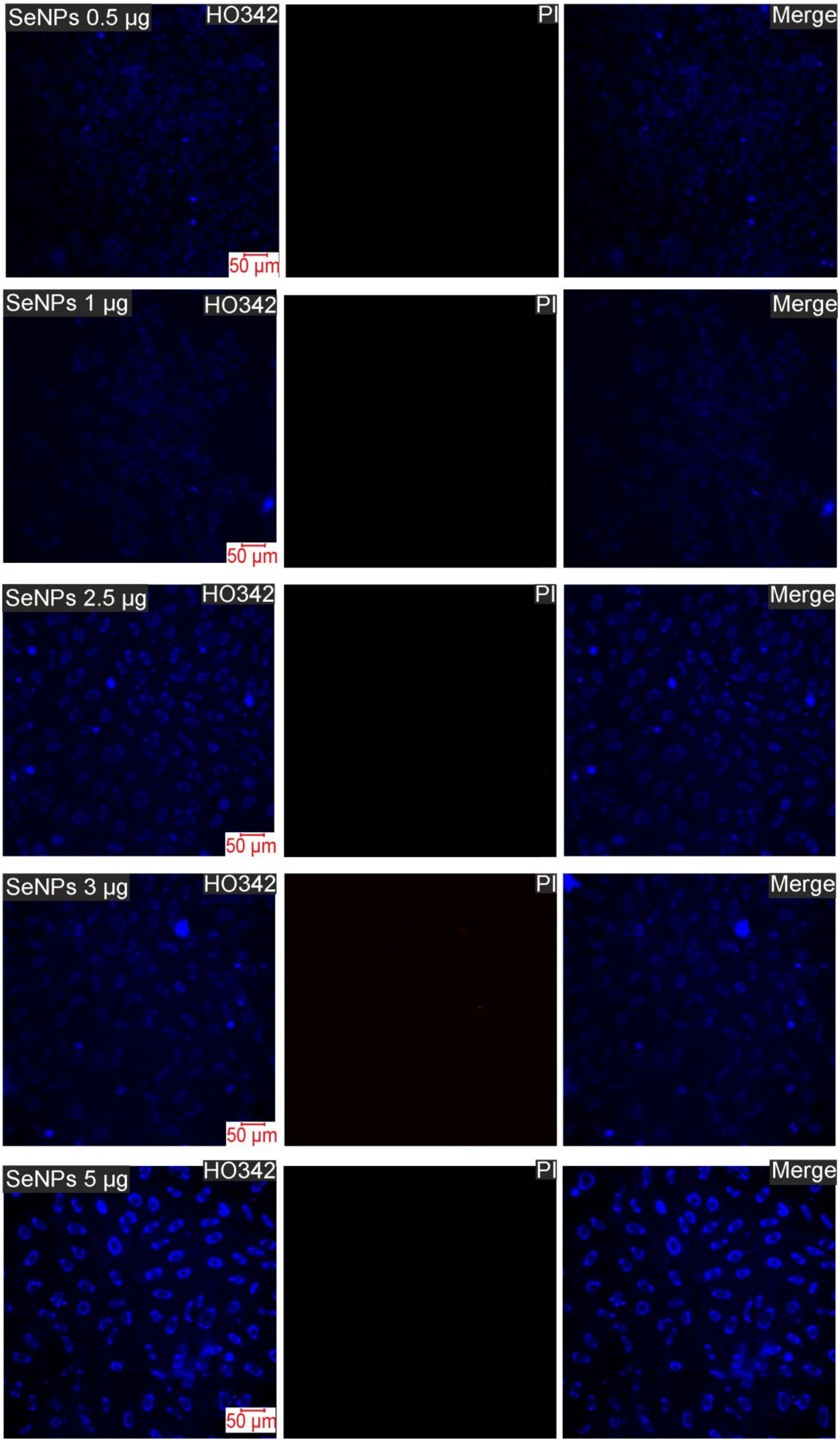

SUPPLEMENTARY, FIGURE S2. Induction of necrosis and apoptosis in the A-172 cells after 24 hour incubation with various 0.5, 1, 2.5, 3 and 5 µg/mL concentrations of 50 nm selenium nanoparticles (SeNPs). Double staining of cells with Hoechst 33342 (HO342), Propidium iodide (PI) and merge (Merge). The images shown in the figure correspond to the data in figure 2 of the text of the manuscript.

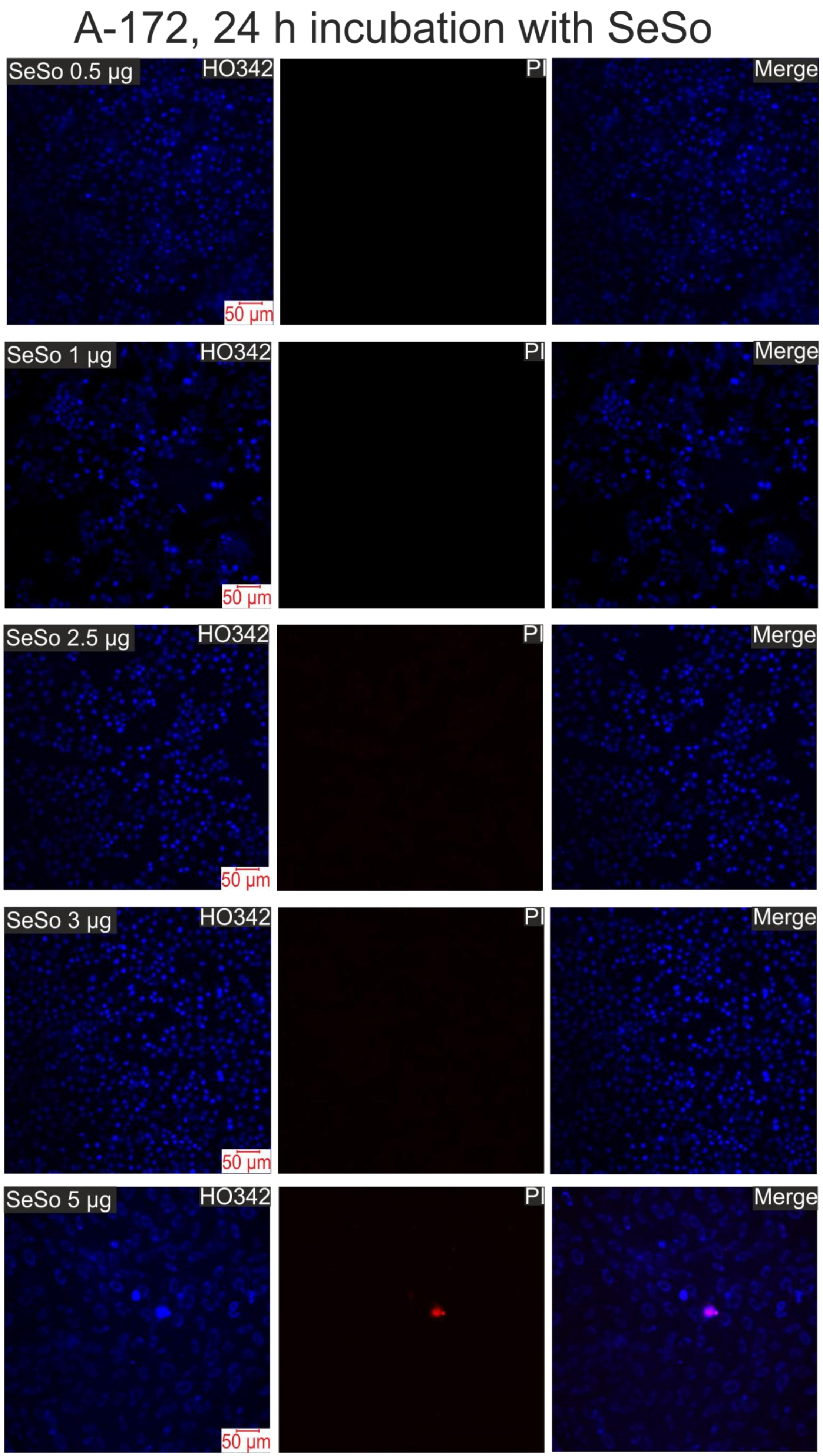
A-172, 24 h incubation with SeSo
SeSo 0.5 µg
HO342
PI
Merge
50 µm
SeSo 1 µg
HO342
PI
Merge
50 µm
SeSo 2.5 µg
HO342
PI
Merge
50 µm
SeSo 3 µg
HO342
PI
Merge
50 µm
SeSo 5 µg
HO342
PI
Merge
50 µm

SUPPLEMENTARY, FIGURE S3. Induction of necrosis and apoptosis in the A-172 cells after 24 hour incubation with various 0.5, 1, 2.5, 3 and 5 μg/mL concentrations of 50 nm selenium nanoparticles doped with sorafenib (SeSo). Double staining of cells with Hoechst 33342 (HO342), Propidium iodide (PI) and merge (Merge). The images shown in the figure correspond to the data in figure 2 of the text of the manuscript.

A-172, 48 h incubation with So

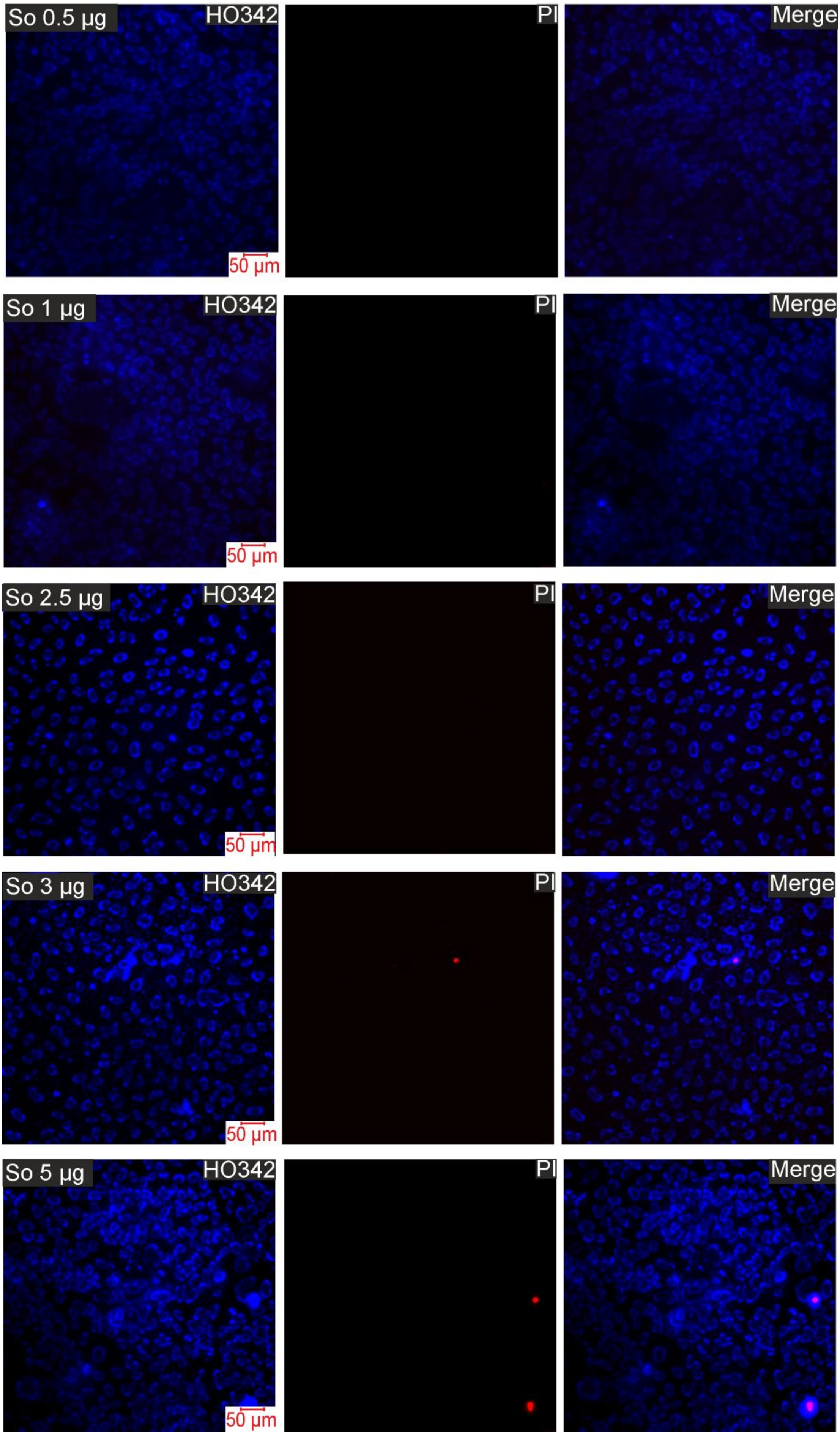

SUPPLEMENTARY, FIGURE S4. Induction of necrosis and apoptosis in the A-172 cells after 48 hour incubation with various 0.5, 1, 2.5, 3 and 5 μg/mL concentrations of sorafenib (So). Double staining of cells with Hoechst 33342 (HO342), Propidium iodide (PI) and merge (Merge). The images shown in the figure correspond to the data in figure 3 of the text of the manuscript.

A-172, 48 h incubation with SeNPs

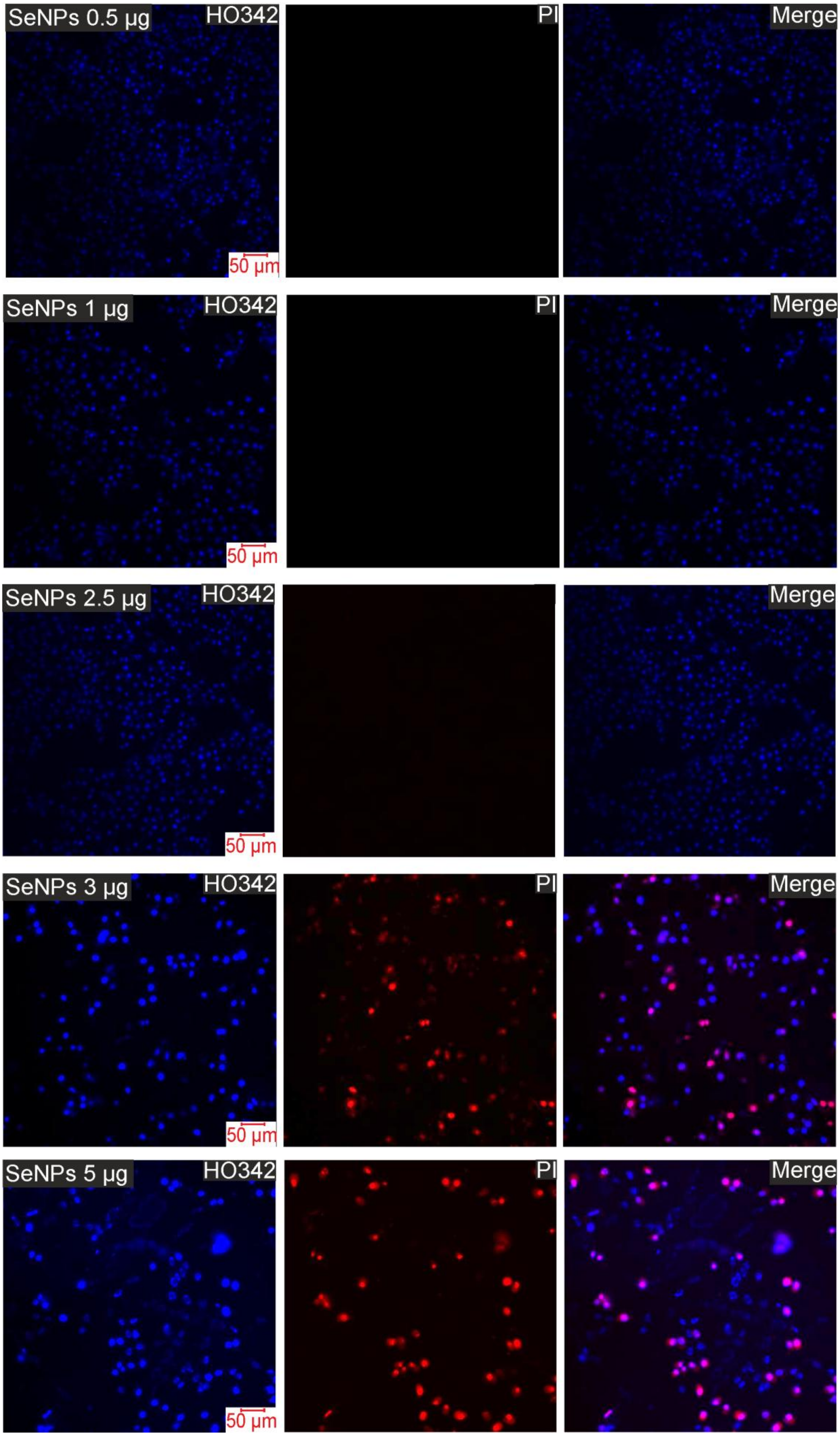

SUPPLEMENTARY, FIGURE S5. Induction of necrosis and apoptosis in the A-172 cells after 48 hour incubation with various 0.5, 1, 2.5, 3 and 5 μg/mL concentrations of 50 nm selenium nanoparticles (SeNPs). Double staining of cells with Hoechst 33342 (HO342), Propidium iodide (PI) and merge (Merge). The images shown in the figure correspond to the data in figure 3 of the text of the manuscript.

A-172, 48 h incubation with SeSo

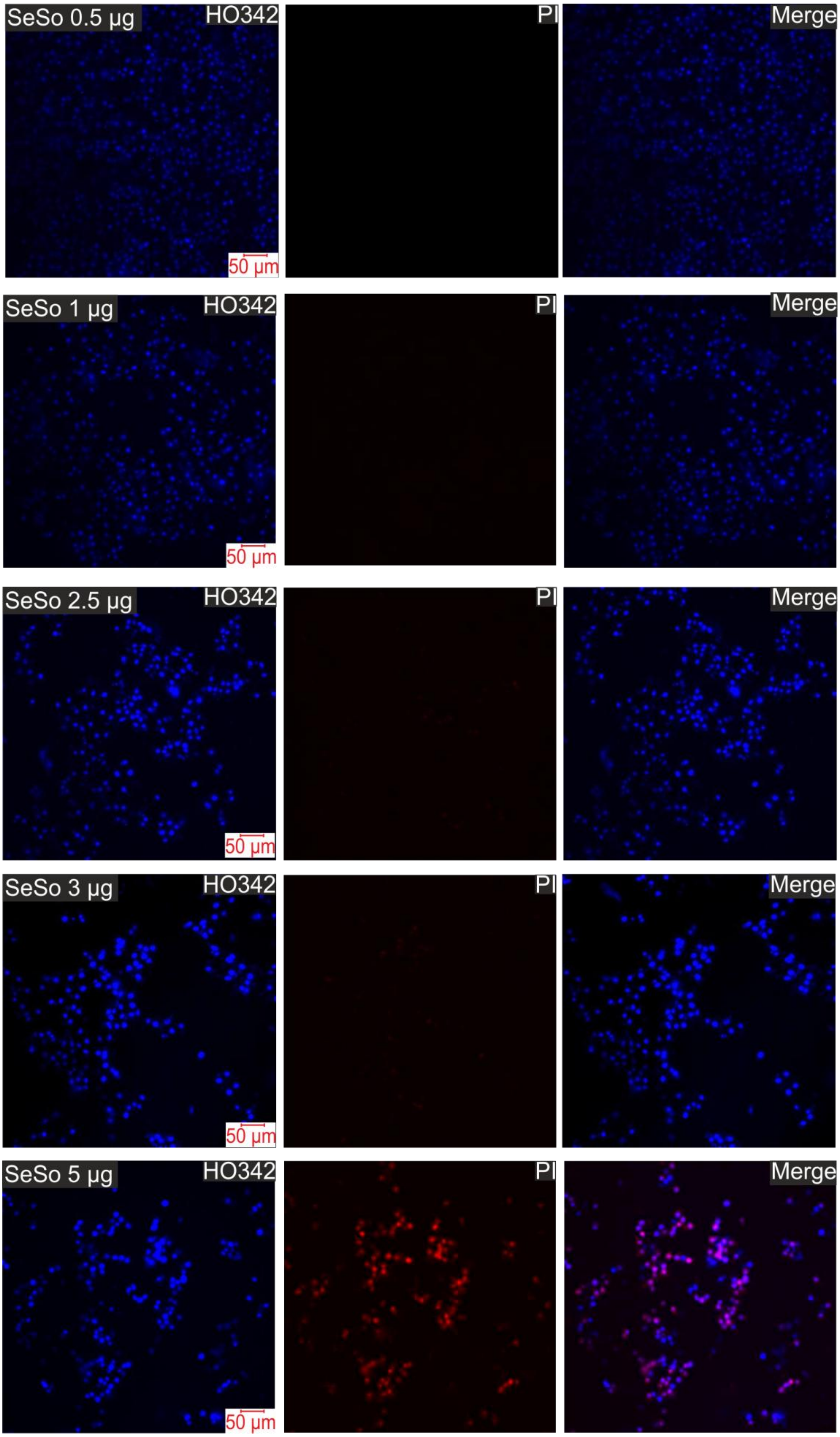

SUPPLEMENTARY, FIGURE S6. Induction of necrosis and apoptosis in the A-172 cells after 48 hour incubation with various 0.5, 1, 2.5, 3 and 5 μg/mL concentrations of 50 nm selenium nanoparticles doped with sorafenib (SeSo). Double staining of cells with Hoechst 33342 (HO342), Propidium iodide (PI) and merge (Merge). The images shown in the figure correspond to the data in figure 3 of the text of the manuscript.

Astrocytes, 24 h incubation with So

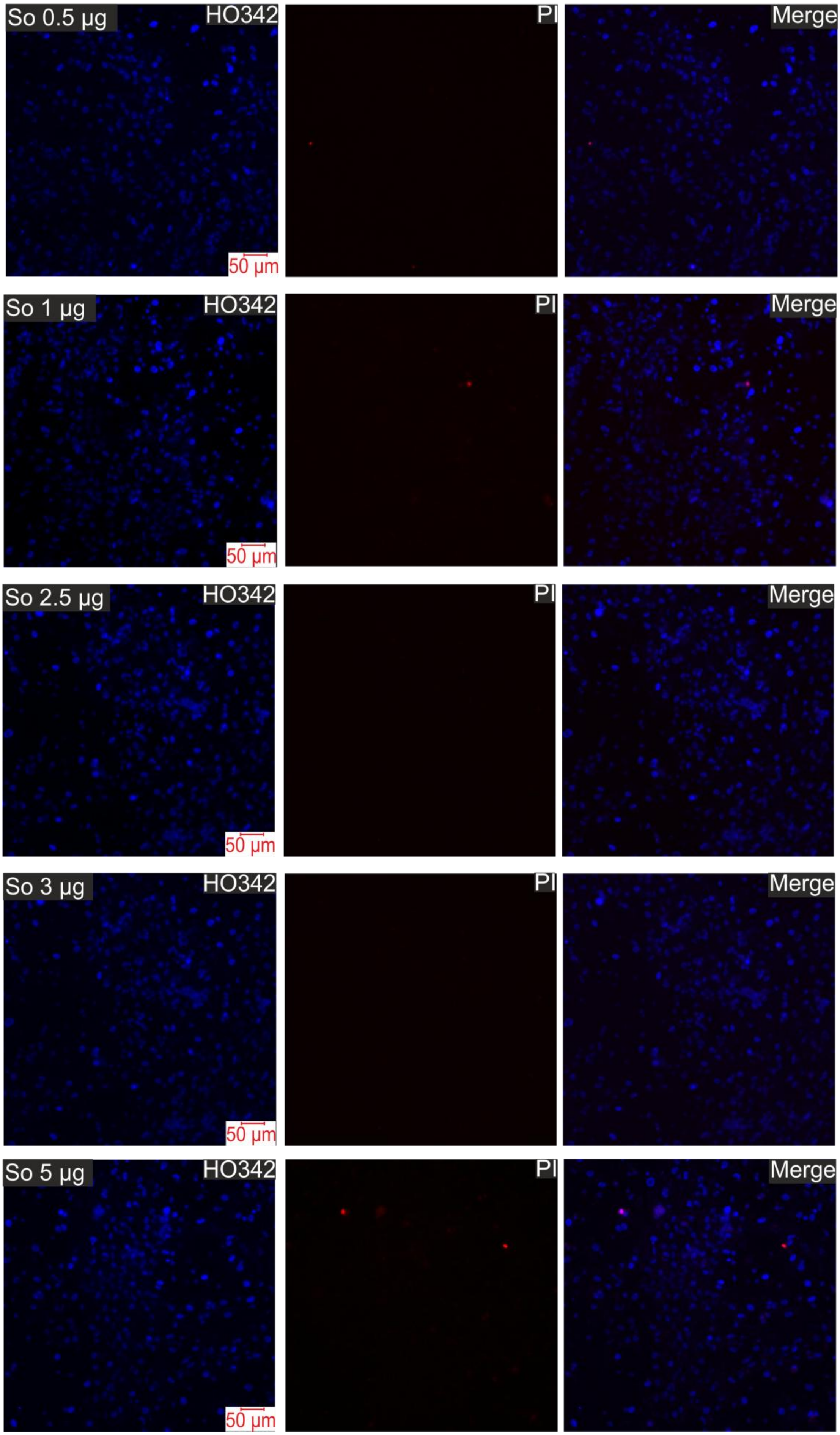

SUPPLEMENTARY, FIGURE S7. Induction of necrosis and apoptosis in the cortical astrocytes after 24 hour incubation with various 0.5, 1, 2.5, 3 and 5 µg/mL concentrations of sorafenib (So). Double staining of cells with Hoechst 33342 (HO342), Propidium iodide (PI) and merge (Merge). The images shown in the figure correspond to the data in figure 4 of the text of the manuscript.

Astrocytes, 24 h incubation with SeNPs

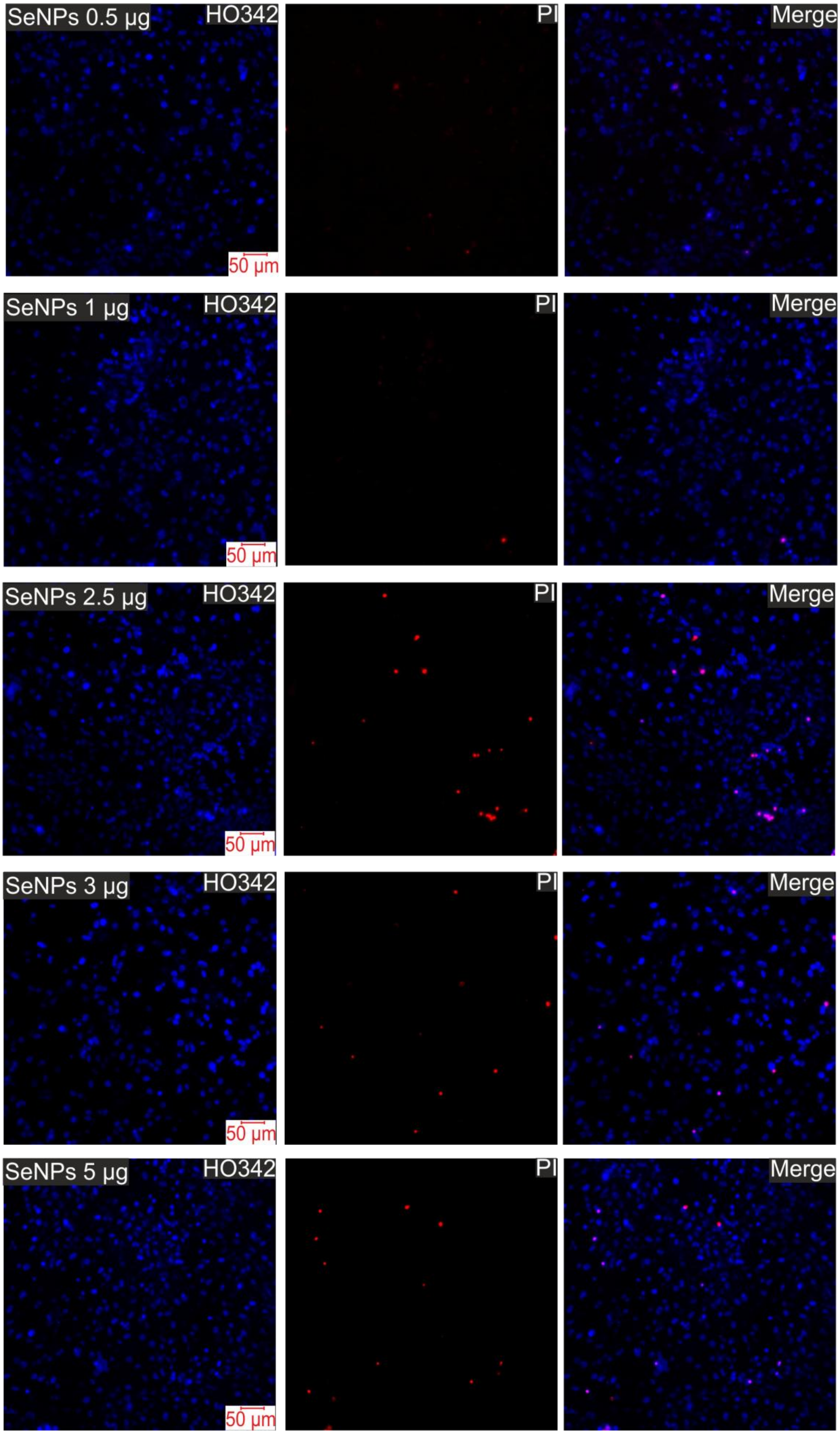

SUPPLEMENTARY, FIGURE S8. Induction of necrosis and apoptosis in the cortical astrocytes after 24 hour incubation with various 0.5, 1, 2.5, 3 and 5 μg/mL concentrations of 50 nm selenium nanoparticles (SeNPs). Double staining of cells with Hoechst 33342 (HO342), Propidium iodide (PI) and merge (Merge). The images shown in the figure correspond to the data in figure 4 of the text of the manuscript.

Astrocytes, 24 h incubation with SeSo

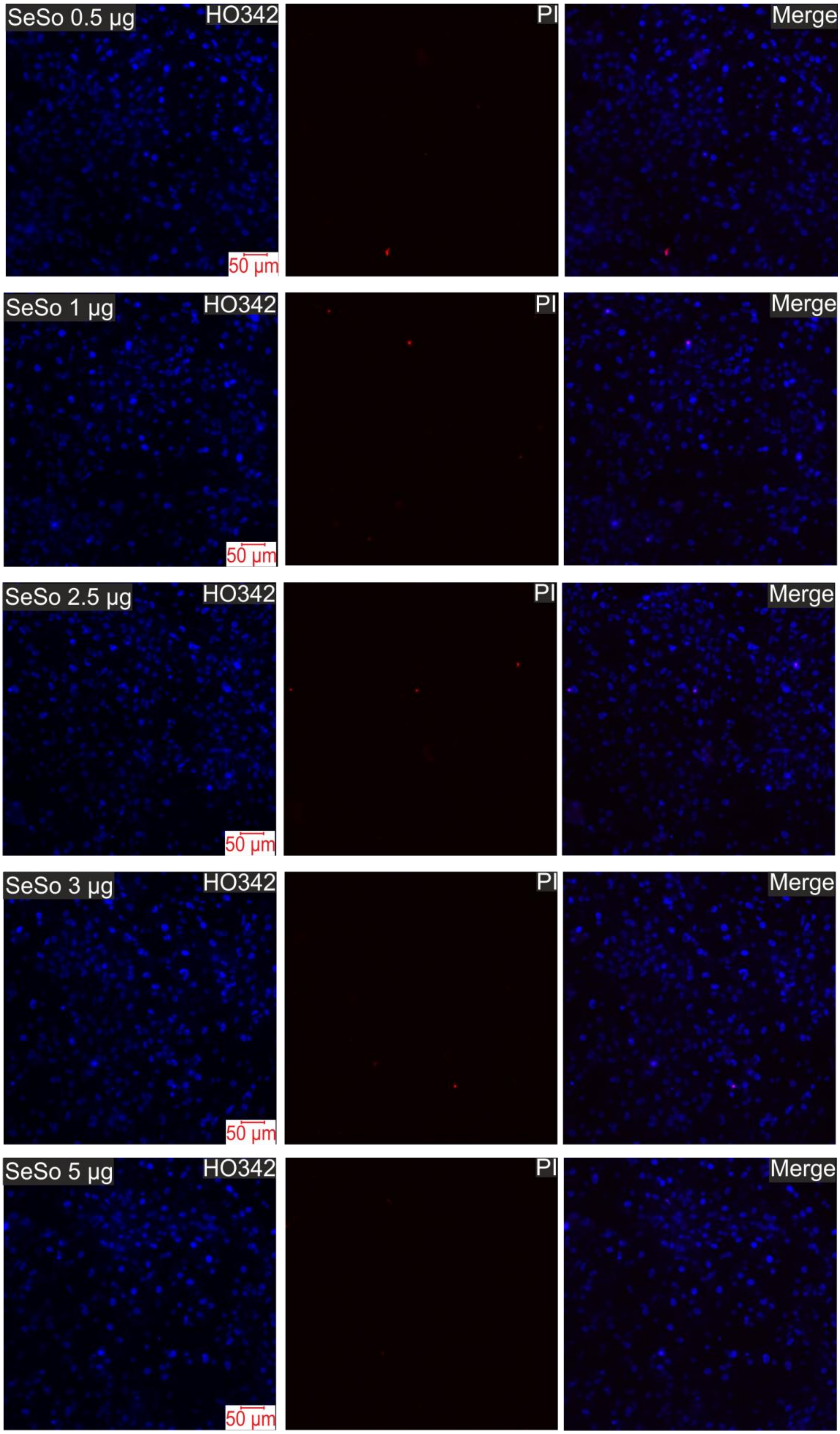

SUPPLEMENTARY, FIGURE S9. Induction of necrosis and apoptosis in the cortical astrocytes after 24 hour incubation with various 0.5, 1, 2.5, 3 and 5 μg/mL concentrations of 50 nm selenium nanoparticles doped with sorafenib (SeSo). Double staining of cells with Hoechst 33342 (HO342), Propidium iodide (PI) and merge (Merge). The images shown in the figure correspond to the data in figure 4 of the text of the manuscript.

Astrocytes, 48 h incubation with So

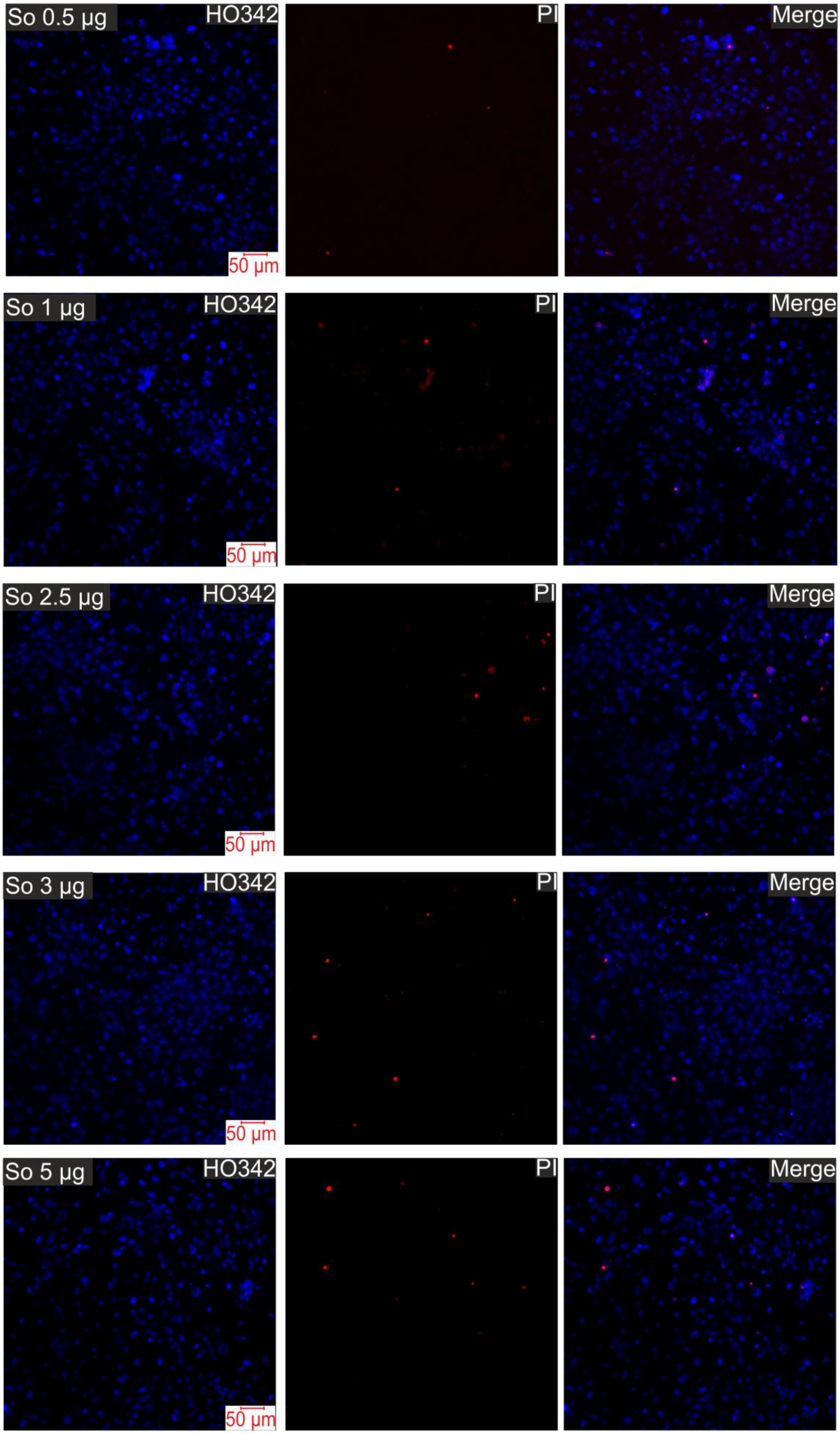

SUPPLEMENTARY, FIGURE S10. Induction of necrosis and apoptosis in the cortical astrocytes after 48 hour incubation with various 0.5, 1, 2.5, 3 and 5 μg/mL concentrations of sorafenib (So). Double staining of cells with Hoechst 33342 (HO342), Propidium iodide (PI) and merge (Merge). The images shown in the figure correspond to the data in figure 5 of the text of the manuscript.

Astrocytes, 48 h incubation with SeNPs

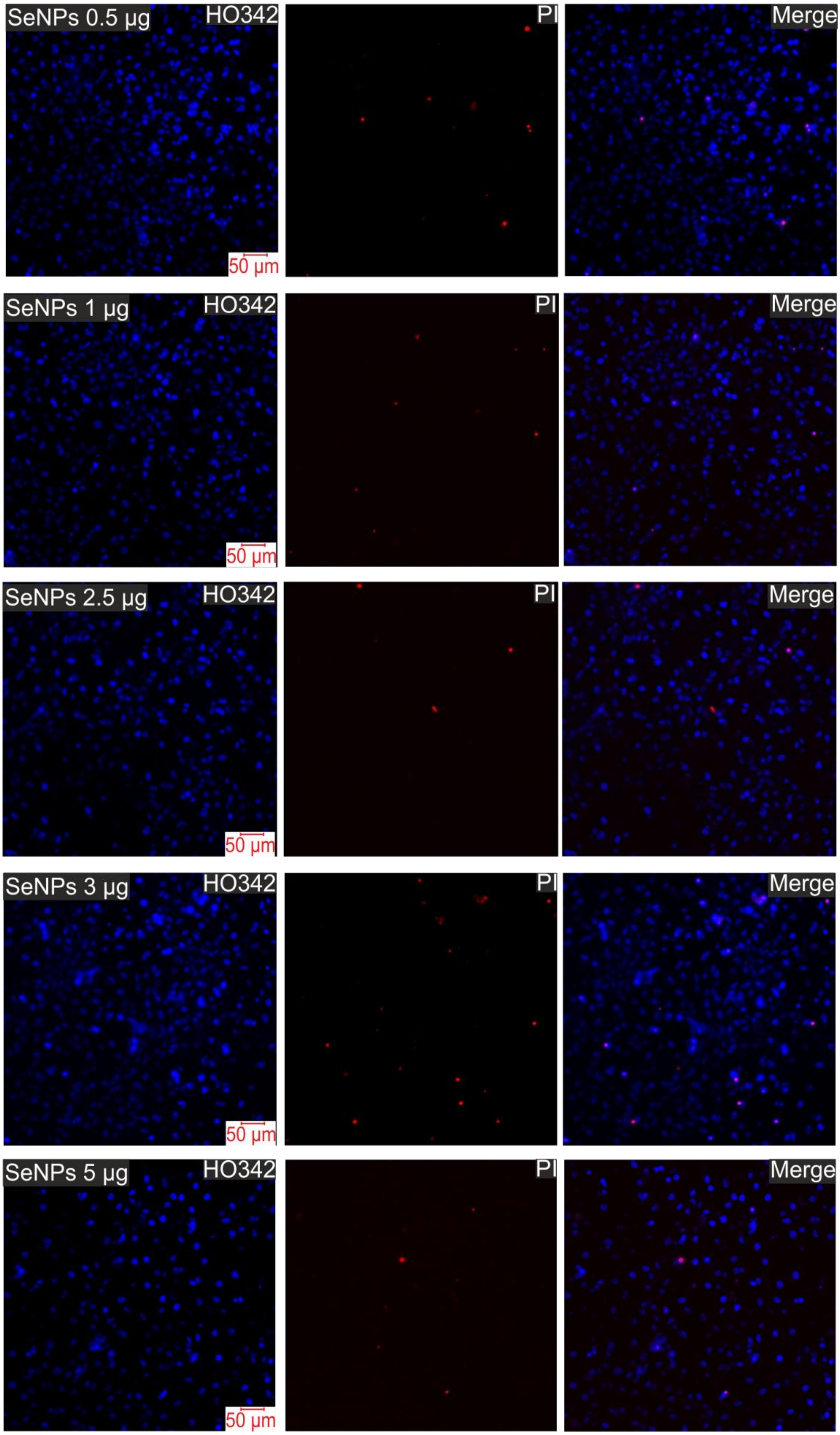

SUPPLEMENTARY, FIGURE S11. Induction of necrosis and apoptosis in the cortical astrocytes after 48 hour incubation with various 0.5, 1, 2.5, 3 and 5 μg/mL concentrations of 50 nm selenium nanoparticles (SeNPs). Double staining of cells with Hoechst 33342 (HO342), Propidium iodide (PI) and merge (Merge). The images shown in the figure correspond to the data in figure 5 of the text of the manuscript.

Astrocytes, 48 h incubation with SeSo

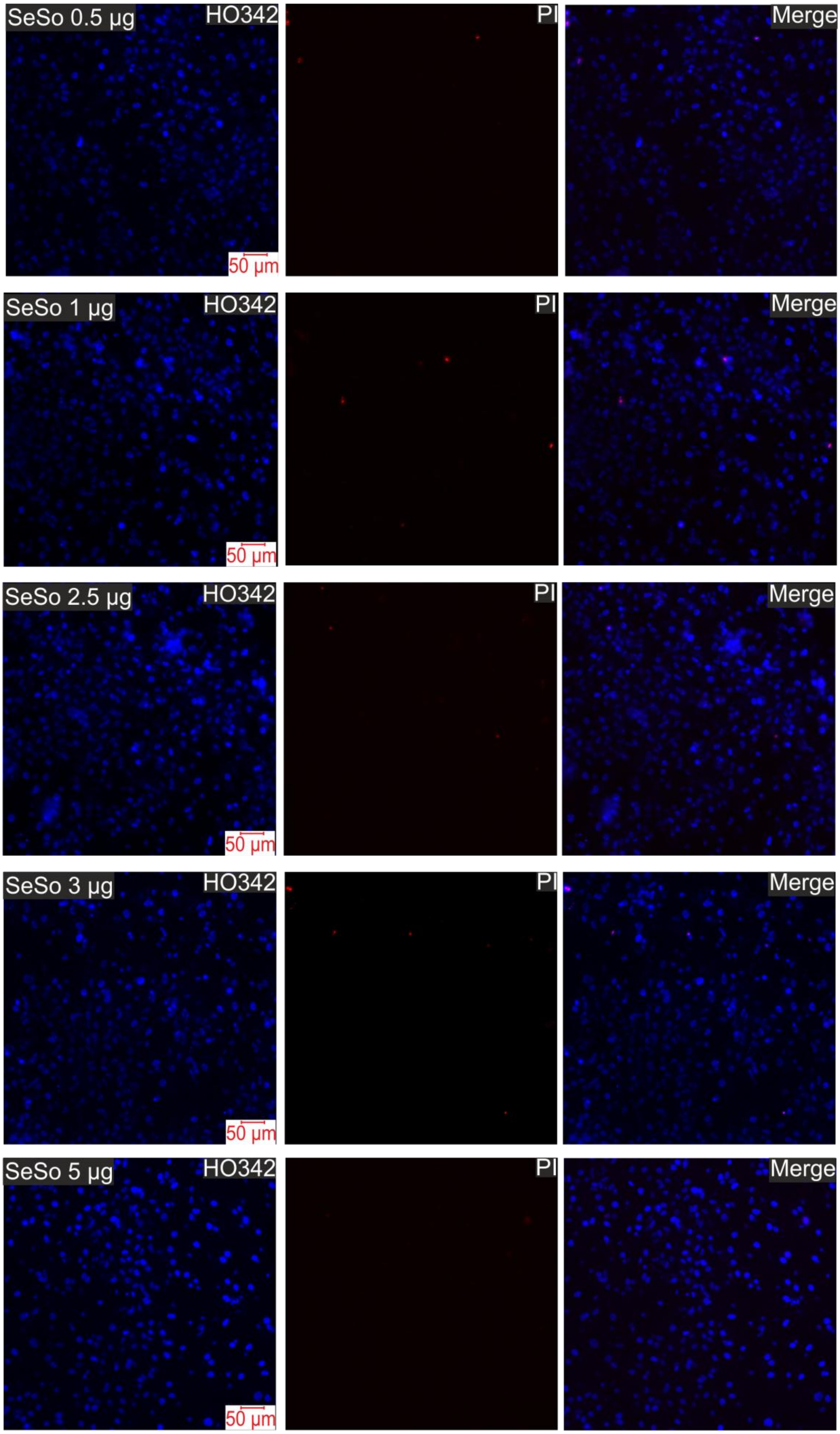

SUPPLEMENTARY, FIGURE S12. Induction of necrosis and apoptosis in the cortical astrocytes after 48 hour incubation with various 0.5, 1, 2.5, 3 and 5 μg/mL concentrations of 50 nm selenium nanoparticles doped with sorafenib (SeSo). Double staining of cells with Hoechst 33342 (HO342), Propidium iodide (PI) and merge (Merge). The images shown in the figure correspond to the data in figure 5 of the text of the manuscript.
